# Vikodak - A Modular Framework for Inferring Functional Potential of Microbial Communities from 16S Metagenomic Datasets

**DOI:** 10.1371/journal.pone.0148347

**Published:** 2016-02-05

**Authors:** Sunil Nagpal, Mohammed Monzoorul Haque, Sharmila S. Mande

**Affiliations:** Bio-Sciences R&D Division, TCS Innovation Labs, Tata Research Development & Design Centre, 54-B, Hadapsar Industrial Estate, Pune 411 013, Maharashtra, India; University of Hyderabad, INDIA

## Abstract

**Background:**

The overall metabolic/functional potential of any given environmental niche is a function of the sum total of genes/proteins/enzymes that are encoded and expressed by various interacting microbes residing in that niche. Consequently, prior (collated) information pertaining to genes, enzymes encoded by the resident microbes can aid in indirectly (re)constructing/ inferring the metabolic/ functional potential of a given microbial community (given its taxonomic abundance profile). In this study, we present Vikodak—a multi-modular package that is based on the above assumption and automates inferring and/ or comparing the functional characteristics of an environment using taxonomic abundance generated from one or more environmental sample datasets. With the underlying assumptions of co-metabolism and independent contributions of different microbes in a community, a concerted effort has been made to accommodate microbial co-existence patterns in various modules incorporated in Vikodak.

**Results:**

Validation experiments on over 1400 metagenomic samples have confirmed the utility of Vikodak in (a) deciphering enzyme abundance profiles of any KEGG metabolic pathway, (b) functional resolution of distinct metagenomic environments, (c) inferring patterns of functional interaction between resident microbes, and (d) automating statistical comparison of functional features of studied microbiomes. Novel features incorporated in Vikodak also facilitate automatic removal of false positives and spurious functional predictions.

**Conclusions:**

With novel provisions for comprehensive functional analysis, inclusion of microbial co-existence pattern based algorithms, automated inter-environment comparisons; in-depth analysis of individual metabolic pathways and greater flexibilities at the user end, Vikodak is expected to be an important value addition to the family of existing tools for 16S based function prediction.

**Availability and Implementation:**

A web implementation of Vikodak can be publicly accessed at: http://metagenomics.atc.tcs.com/vikodak. This web service is freely available for all categories of users (academic as well as commercial).

## Introduction

The field of metagenomics has significantly improved our overall understanding of the microbial world within and around us. Characterizing microbial communities using the metagenomics approach involves either (a) performing a whole genome (shotgun) sequencing (referred to as WGS) of the entire genomic content of a given environmental sample, or (b) performing amplicon sequencing of specific marker genes (e.g. 16S rRNA) from any microbial community. The focus/end-objectives of the research problem (as well as associated sequencing costs) typically drive the choice between adopting a WGS and a 16S amplicon sequencing approach. The 16S approach specifically helps in deciphering the taxonomic/microbial composition of a given environmental sample, thereby paving the way for performing specific diversity analysis and subsequent identification of environment-specific marker microbes. The WGS approach, on the other hand, offers a two-fold advantage. Besides helping in obtaining a taxonomic profile of the environmental sample under study, computational analysis of WGS data provides information at a functional level (i.e. types and relative abundances of genes encoded by various microbes in a given environment). Given the relatively higher costs associated with WGS sequencing (and the computational complexity of handling huge volumes of WGS data), a majority of metagenomic initiatives employ 16S sequencing for obtaining a quick comparative snap-shot of microbial diversity. Subsequently, a WGS follow-up experiment is sometimes performed (on a relatively smaller subset of samples), typically when 16S experiments indicate significant aberrations (in microbial composition) in the analysed sample classes (e.g. between healthy and diseased states).

Metagenomic data analysis of microbial communities is typically aimed at (a) identifying the resident microbes and their relative proportions (i.e. taxonomic analysis), (b) profiling the functions encoded by these microbes (i.e. functional annotation), and (c) comparing/ co-relating the identified microbes and their functions with available sampling (phenotypic) metadata (i.e. comparative metagenomics). Several stand-alone tools and web-services are available for performing the above mentioned analyses [1–5]. Currently, the number of existing tools/ platforms for functional characterization of a metagenomic environment is relatively lower that that available for taxonomic analysis and comparative metagenomics. This scenario is however changing due to the emergence (and successful validation) of a new line of thought that proposes inferring/ (re)constructing the metabolic/ functional potential (of a given environmental sample) using its 16S taxonomic abundance profile. The rationale behind this line of thought is based on the fact that the overall metabolic/ functional potential of any given environmental niche is a function of the sum total of genes/ proteins/ enzymes that are encoded by various microbes residing in that niche. Consequently, a pre-computed database containing collated information pertaining to genes, enzymes encoded by various microbes (known till date) can potentially be employed for indirectly inferring/ (re)constructing the metabolic/ functional potential of a microbial community based on its 16S taxonomic abundance profile.

The idea of inferring the functional potential using 16S-derived microbial abundance data has been attempted by a handful of research groups. For example, approaches like procrustes analysis [6] and ancestral-state reconstruction [2] have been successfully applied (and validated) for predicting functional potential of a microbial community from its 16S rRNA sequence data. Tools like METAGENEassist [4] have furthered this premise by extending the idea to prediction of phenotypic traits and physiological functions such as pH, oxygen requirement etc., of an environmental sample by analysing its microbial composition.

Functional characterization of any metagenomic sample should ideally enable insights into (i) functional units (genes/ enzymes) expressed by resident microbes, (ii) relative abundance and expression levels of various metabolic pathways, (iii) relationships between various resident microbes based upon their individual functional characteristics, (iv) core functions of an environment, (v) differences between distinct environments, and (vi) genes/ enzymes associated with individual functions (viz. metabolic pathways). In addition, the functional profile should be devoid of functions specific to eukaryotes. While the first two objectives of an ideal functional characterisation are fulfilled by the conventional WGS-based functional annotation tools [3,5] and the recently developed 16S-based functional prediction algorithms [1,2], a majority of other objectives have not been addressed in the current state-of-art.

In this study, we describe Vikodak (*decoder* in Sanskrit)—a modular functional annotation tool that extends the functionality/ utility of the '16S-data-inferred-function-prediction' paradigm. A detailed description of (a) various modules of Vikodak (and the algorithmic work-flow they employ), and (b) additional features incorporated in Vikodak that address the unmet objectives of existing 16S-based functional prediction algorithms is provided. In addition to the validation of results obtained with greater than 1400 metagenomic samples, a case study (performed using publicly available Periodontitis metagenomics datasets) that highlights the functional utility of various modules incorporated in Vikodak is also presented.

## Methods

Vikodak contains three distinct functional modules viz. Global Mapper, Inter Sample Feature Analyzer (ISFA), and Local Mapper, each catering to specific end-user requirements. Briefly, given a microbial abundance data profile of an environmental sample, the Global Mapper module enables (a) an *in silico* estimation of the relative abundance of various metabolic pathways in that sample, (b) quantifying the contribution of each of the microbes (in that sample) to the predicted functions (at all three tiers of KEGG [7] hierarchy), and (c) identification of the core set of metabolic functions defining a particular environment. The ISFA module in Vikodak is a logical extension of the Global Mapper module and is designed for performing a rigorous (pair-wise) comparative statistical analysis of the (inferred/ predicted) functional profiles generated from two or more environments. The Local Mapper module further enables end-users to probe, in greater detail, the enzyme abundance profile(s) of individual metabolic pathway(s) identified as (a) the 'core' in one or more environments, or (b) differentially abundant between two or more environments. It is pertinent to note here that end-users can, in principle, employ Local Mapper module for analysing the enzyme abundance profile of any metabolic pathway (and not just the 'core' or differentially abundant pathways). In spite of adopting a distinct algorithmic work-flow, all three modules described above share the same (pre-built) back-end database. Details of (a) the procedure followed for building the back-end database, and (b) detailed description of the algorithm/ work-flow adopted in the three modules of Vikodak are provided below.

### A. Vikodak's Back-end Database

The overall functional characteristics of any microbial community are defined by the metabolic potential of the resident microbes. This, in turn, is dependent on the presence and abundance pattern of various genes/ enzymes encoded by these microbes. Given this, a pre-compiled database containing all available information about various enzymes and their copy numbers (an index of the magnitude of expression) in various microbes is expected to indirectly enable prediction of the functional traits/ metabolic potential of a given community of microbes constituting an environmental niche.

In line with the above principle, Vikodak's back-end database was constructed in the following manner. The EC profiles of 12,260 prokaryotic genomes from the PATRIC database [8] and 'Enzyme Abundance Profiles' i.e. gene copy number data of 25,060 prokaryotic genomes from the IMG database [9] were downloaded and suitably parsed to compile mean copy number information for 2,600 enzymes at various levels of taxonomic hierarchy. The choice of using ‘mean’ copy numbers was validated by analysing z-score divergence of copy numbers corresponding to individual enzymes(in all 37,320 prokaryotic genomes)at various taxonomic levels viz. genus, family, order, class and phylum. Results of this analysis (S1 Fig) indicated that greater than 90–95% of enzyme copy numbers exhibited a z-score range of -1 to +1 thereby suggesting the appropriateness of using ‘mean’ copy numbers at higher taxonomic levels (including phylum). Furthermore, an enzyme to KEGG Pathway Mapping Database (at all three tiers of KEGG hierarchy) was compiled by using three hash-maps. S2 Fig gives a schematic representation of the three levels of KEGG pathway hierarchy and corresponding hash-maps. Overall, the hash-maps contained information about 3275 enzymes, 360 KEGG Pathways (Level 3), 42 KEGG Pathway classes (Level 2) and 6 KEGG Pathway super-classes (Level 1).

### B. Algorithm(s) adopted in the three modules of Vikodak

#### 1. Global Mapper

**Fig 1:**
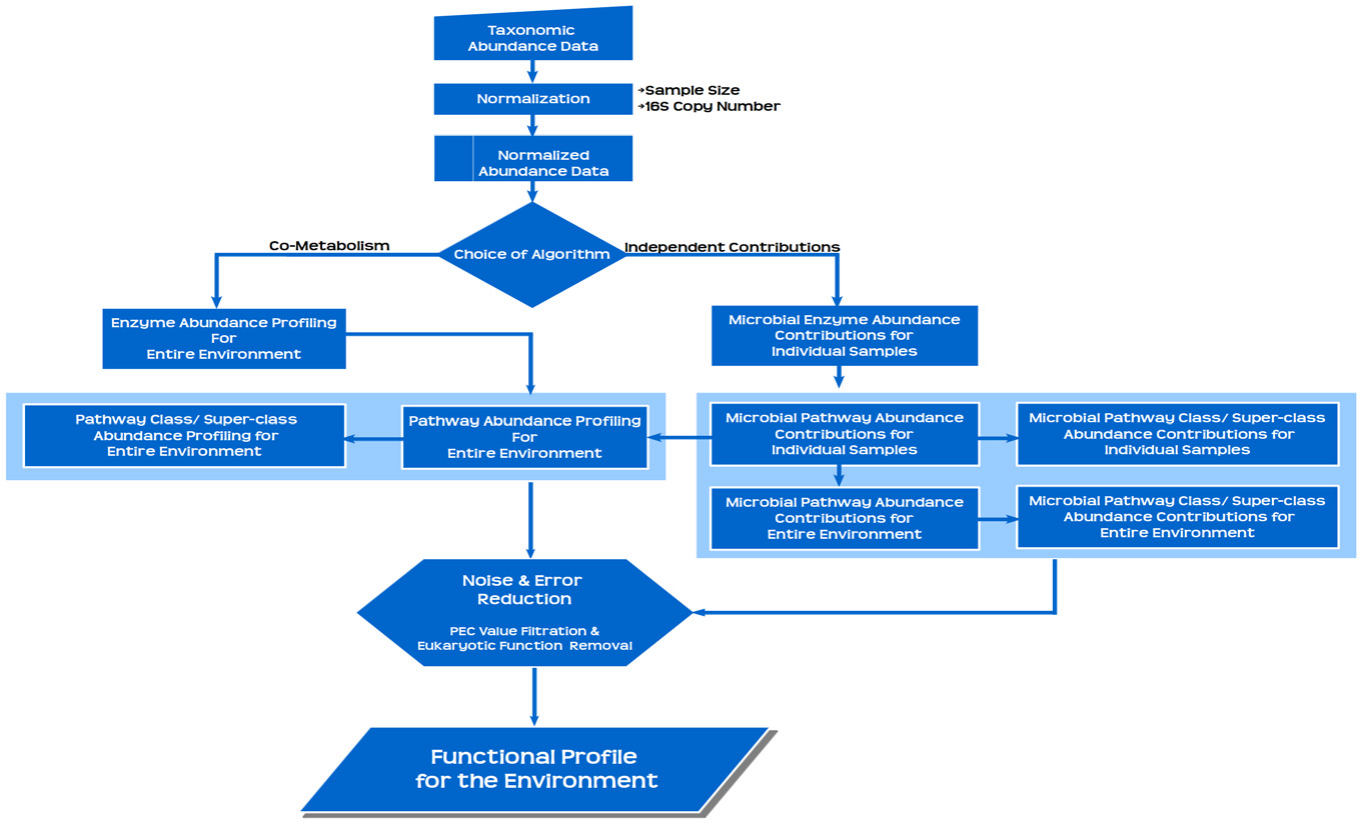
Global Mapper Workflow. A flowchart depicting the overall workflow of Global Mapper module of Vikodak. This module enables users to computationally estimate the relative abundance of various metabolic pathways in a given sample/ environment. It helps in quantifying the contribution of individual microbes (in that sample) to the predicted functions at all three tiers of KEGG hierarchy. These functionalities are achieved using two distinct algorithmic workflows, viz. co-metabolism and independent contributions. For filtering out spuriously predicted functions, the module incorporates two mechanisms, namely, Pathway Exclusion Cut-off (PEC) and ‘Removal of eukaryotic functions’.

A set of microbes in an environment can either exist as a consortium (i.e. with co-metabolic associations) or they might thrive independently. Considering these two possibilities (of bacterial co-existence), Global Mapper provides to end-users the choice of two distinct algorithmic work-flows viz. 'Co-metabolism' and 'Independent Contributions'. The principle behind these two work-flows is as follows. The co-metabolism algorithm is based on the underlying assumption that the genes/ enzymes expressed by various microbes residing in an environment may pool together and contribute to the functioning of specific metabolic pathway(s). Therefore, the effective abundance of a metabolic pathway (in an environment with co-metabolising microbes) is expected to be a function of the total enzyme pool contributed by the co-metabolising microbes. In contrast, the 'independent contributions' algorithm assumes the independent existence of microbes in the environment. Under this assumption, the effective abundance of a metabolic pathway is the sum total of the pathway abundances computed from individual microbes residing in that environment. S3 Fig gives a schematic description of the assumptions underlying the two algorithms of Global Mapper. A description of the detailed algorithmic work-flow adopted by both sub-modules of Global Mapper is provided in Section A of S1 File. Fig 1 represents a flowchart depicting the overall workflow of Global Mapper. S4 Fig gives a schematic representation of the work-flow and the format of various outputs generated using this module. Various types of analyses that can be performed using the generated outputs are also indicated.

In addition to computing 'results' pertaining to the relative abundance of various metabolic pathways and identifying 'core' metabolic functions, the Global Mapper module incorporates two output refinement mechanisms that improve the overall confidence of the predicted/ inferred functions (Section B of S1 File). The procedure and the rationale behind the use of these refinement mechanisms are described below.

#### (a) Pathway Exclusion Cut-off value (PEC value)

The functioning of a metabolic pathway is dependent on the presence of a minimum number of constituent genes/ enzymes. Consequently, it is crucial to define a suitable threshold value (w. r. t. the proportion of constituent genes/ enzymes) for reporting a pathway to be present/ functional. This threshold is referred to as 'PEC value' (acronym for Pathway Exclusion cut-off Value) in Vikodak. Both sub-modules of Global Mapper incorporate this parameter and provide to end-users multiple results that report 'pathway abundance profiles' (at all three level levels of KEGG hierarchy) for 7 values of PEC (viz. 30, 40, 50, 60, 70, 80 and 90). The utility of this refinement parameter is further highlighted in results (section: Relevance of PEC value).

#### (b) Removal of Eukaryotic Functions

Considering that functional analysis is being performed with an objective of studying functions encoded by the constituent 'microbes' (of an environment), it is logical to exclude pathways that are specific to and are associated with eukaryotic genomes from the final predictions. For this purpose, a list of Eukaryote-associated pathways was compiled by mining literature from public resources as well as from information available in the KEGG database. The mined list includes functions/ KEGG pathways like MAPK signalling pathway, Oocyte meiosis, Plant hormone signal transduction, Rheumatoid arthritis, Vascular smooth muscle contraction, etc. Vikodak provides this refinement procedure as an additional option to end-users.

#### 2. Inter Sample Feature Analyzer (ISFA)

This module automates the statistical comparison of (Global Mapper-derived) functional profiles of two environmental samples. The input data for ISFA is a union of functional profiles (viz. KEGG pathway abundance data or pathway class/ super-class abundance data) of two different environments (sought to be compared). The module employs a 'bootstrapped' approach for statistical comparison wherein a random subset of samples (from both environments) are drawn multiple times and Wilcoxon rank sum test is (performed on sample sets drawn in each iteration) used for identifying functions (pathways/ pathway classes/ pathway super-classes) with significantly different abundances (end-user specified p-value) in each iteration. The ISFA module reports a function as significantly different between two environments only when it is identified as significantly different in a minimum percentage of iterations (the 'boot-strap' threshold/ percentage being specified by the end-user). The end-user is also given the option to determine the number of iterations as well as the size of the random subset (to be drawn in each iteration). S2 File (section H) represents an example of the input and output files required/ generated by ISFA.

#### 3. Local Mapper

This module is a logical extension of the co-metabolism sub-module (of Global Mapper) and the ISFA module of Vikodak. Local Mapper module enables end-users to compute the relative abundance of enzymes constituting a specific pathway of interest (in one or more environmental samples). Except for the usage of back-end hash-maps specific only to the end-user specified pathway, the overall strategy adopted by Local Mapper (for computing enzyme abundances) is identical to that used in Co-metabolism sub-module (please refer S1 File). For comparing the enzyme abundances (of a specific pathway) between two or more environments, Local Mapper rank normalizes the computed abundances and provides this information in the form of a 'user data mapping file'. The format of this mapping file conforms to the input required by the KEGG web-server for graphically visualizing relative enzyme abundances. S2 File (section I) provides the format of the input data-types acceptable in Local Mapper module. S2 File (section J) and S5 Fig contain sample outputs generated by this module.

## Results

Evaluation of various functionalities of Vikodak was performed considering the following questions/ objectives -

1. Is there a concurrence between Vikodak derived functional inferences (from 16S datasets) and those generated from Whole Genome Sequencing (WGS) datasets (generated from the same environment)?
2. Can Vikodak distinguish between datasets taken from environments that are known/ expected to be functionally diverse? How does Vikodak compare with PICRUSt [2] in this aspect?
3. Evaluating the utility of Vikodak's 'Pathway Exclusion Cut-off (PEC)' parameter.
4. A case study highlighting the biological relevance of Vikodak's functional predictions.

### Concurrence of Vikodak's predictions with WGS-derived functions

For estimating concurrence between functional profiles inferred from 16S data (using Vikodak) and WGS-derived functions, 16S taxonomic profiles and WGS-derived functional profiles corresponding to 103 gut metagenomes were downloaded from the MG-RAST [5] database (accession numbers: ‘qiime:850’ and ‘qiime:621’). The 16S taxonomic profiles were provided as input to Vikodak's Global mapper module for obtaining/ inferring corresponding functional profiles. Pearson correlation coefficients between WGS-derived and 16S-derived functional profiles were computed for estimating concurrence between corresponding profiles (for all 103 samples). Vikodak's functional predictions indicate a fair degree of correlation (mean Pearson correlation coefficient value = 0.73, p<0.001) with the WGS-derived functional profiles. The degree of correlation is even higher (mean Pearson correlation coefficient value = 0.97, p<0.001) when the eukaryotic function(s) within the generated functional profiles are filtered out using the option provided in Vikodak. These results indicate that Vikodak can enable end-users to predict/ infer (from 16S data) the functional profile (of a given environment) which is fairly equivalent to that obtained by employing a WGS-based experimental approach.

### Segregation of environmental datasets based on Vikodak derived functions

**Fig 2:**
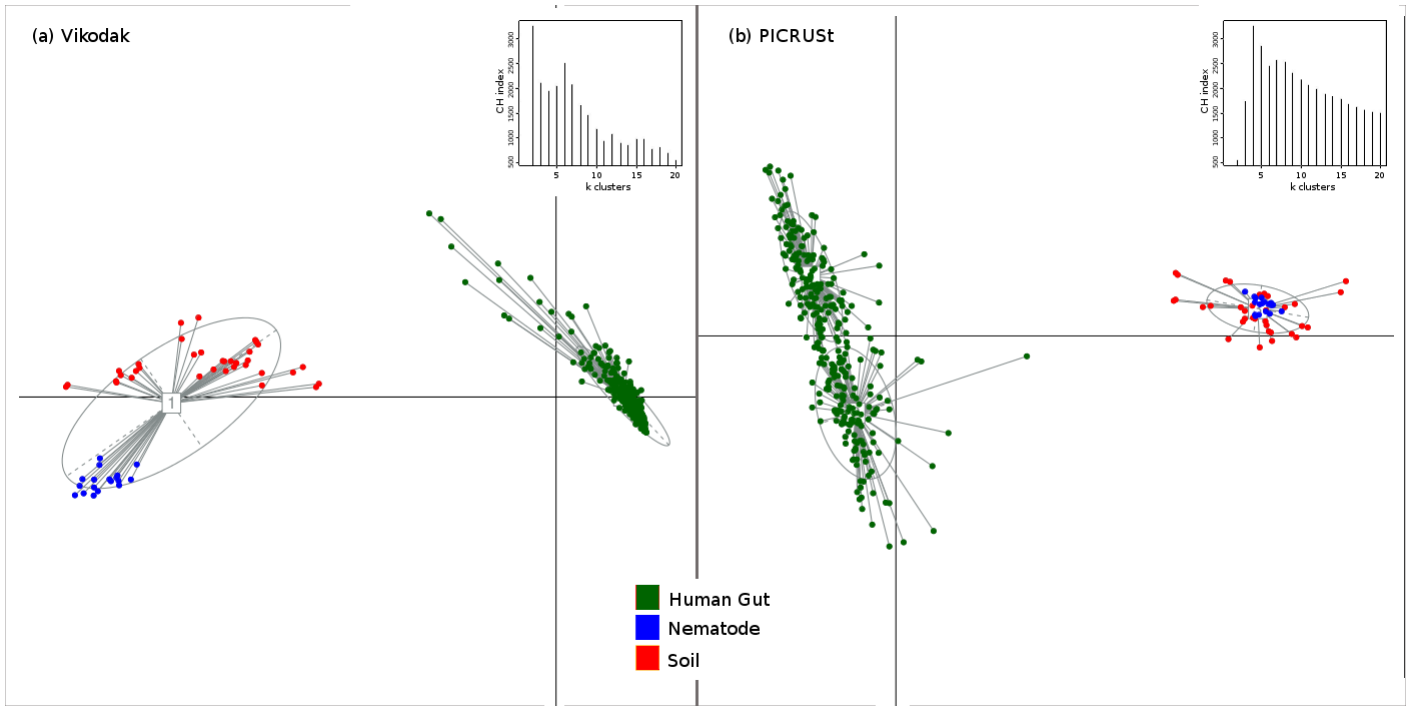
Comparison of Vikodak with PICRUSt using datasets from distinct environments. JSD based PCoA ordination of functional inferences obtained using (a) Vikodak (b) PICRUStfor 364 samples pertaining to three distinct environments viz. Human Gut, Nematode and Amazonian soil. In spite of adopting different methodologies for inferring functions from 16S datasets, both Vikodak and PICRUSt are able to obtain a distinct spatial clustering of datasets according to the sampling environments. S1 Table may be referred for details pertaining to the sourced metagenomes.

**Fig 3:**
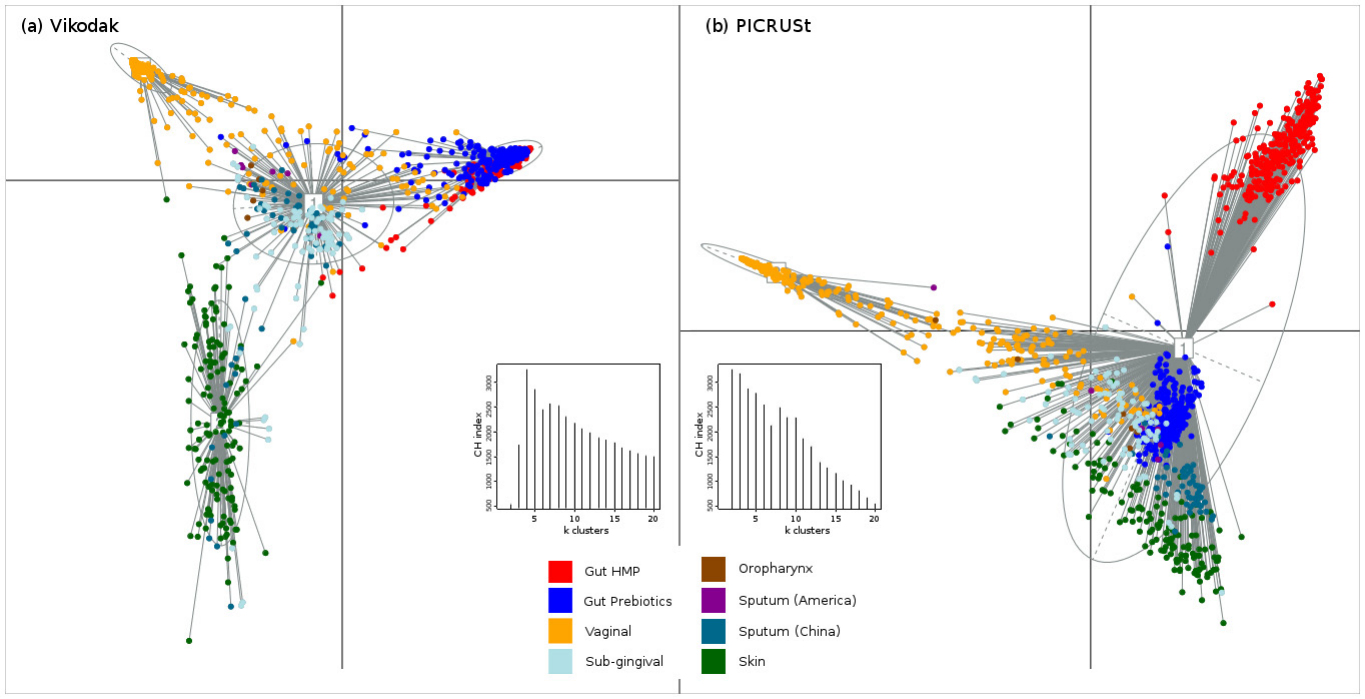
Comparison of Vikodak with PICRUSt using datasets from physiologically distinct body sites in humans. JSD based PCoA ordination of functional inferences obtained using (a) Vikodak (b) PICRUSt for 1291 samples pertaining to four distinct human body sites, viz. Gut (HMP and Prebiotics), Oral Cavity (Oropharynx, Sub-gingival and Sputum), Skin and Vagina. The pattern of clustering indicates notable differences in the performance of Vikodak and PICRUSt. Vikodak-derived functional profiles of physiologically similar environments are observed to exhibit a logical/ expected pattern of clustering as compared to PICRUSt. For instance, Vikodak-derived functional profiles of all gut samples are observed to cluster closely together despite belonging to different experiments (and geographies). A similar pattern of clustering is observed for samples belonging to the oral cavity. S1 Table may be referred for details pertaining to the sourced metagenomes.

In order to evaluate the efficiency/ accuracy of Vikodak in distinguishing between datasets taken from functionally diverse environments, two experiments were performed. In the first experiment, taxonomic abundance data corresponding to 360 (publicly available) metagenomic datasets from three functionally distinct environments was analysed using (a) Global Mapper module of Vikodak (using pathway level data) and (b) the PICRUSt tool (using L3 level data). These three environments included human gut (HMP), *Litoditis marina* nematode species (SRP064694), and Amazonian soil (ERA411828). In the second experiment, taxonomic abundance data corresponding to 1291 samples, pertaining to various human body sites (gut, oral cavity, vagina, and skin), were analysed in a similar manner. S1 Table provides details of all datasets used in the above experiments. PCoA-based ordination analysis of the generated 'Pathway abundance profiles' (in both experiments) was performed using the procedure described in Arumugam *et al*., 2011 [10]. For the first experiment, the ordination plots (Fig 2a and 2b) resulting from both Vikodak and PICRUSt indicate a distinct spatial clustering of datasets [11–18] according to the sampling environments. Results of the second experiment (Fig 3a and 3b), however, indicate notable differences in the performance of both tools. Vikodak-derived functional profiles of physiologically similar environments (e.g. gut and oral cavity) are observed to exhibit a logical/ expected pattern of clustering as compared to PICRUSt. For instance, all gut samples (belonging to the HMP and Pre-biotic datasets) are observed to cluster closely together, even though these datasets belonged to different experiments [11–13] and geographies (America, Japan and China). A similar pattern of clustering is observed for samples belonging to the oral cavity [14,15]. These results indicate Vikodak's efficiency in correctly inferring the functional potential of environments that are known to be physiologically diverse.

### Relevance of Pathway Exclusion Cut-off (PEC) value

**Fig 4:**
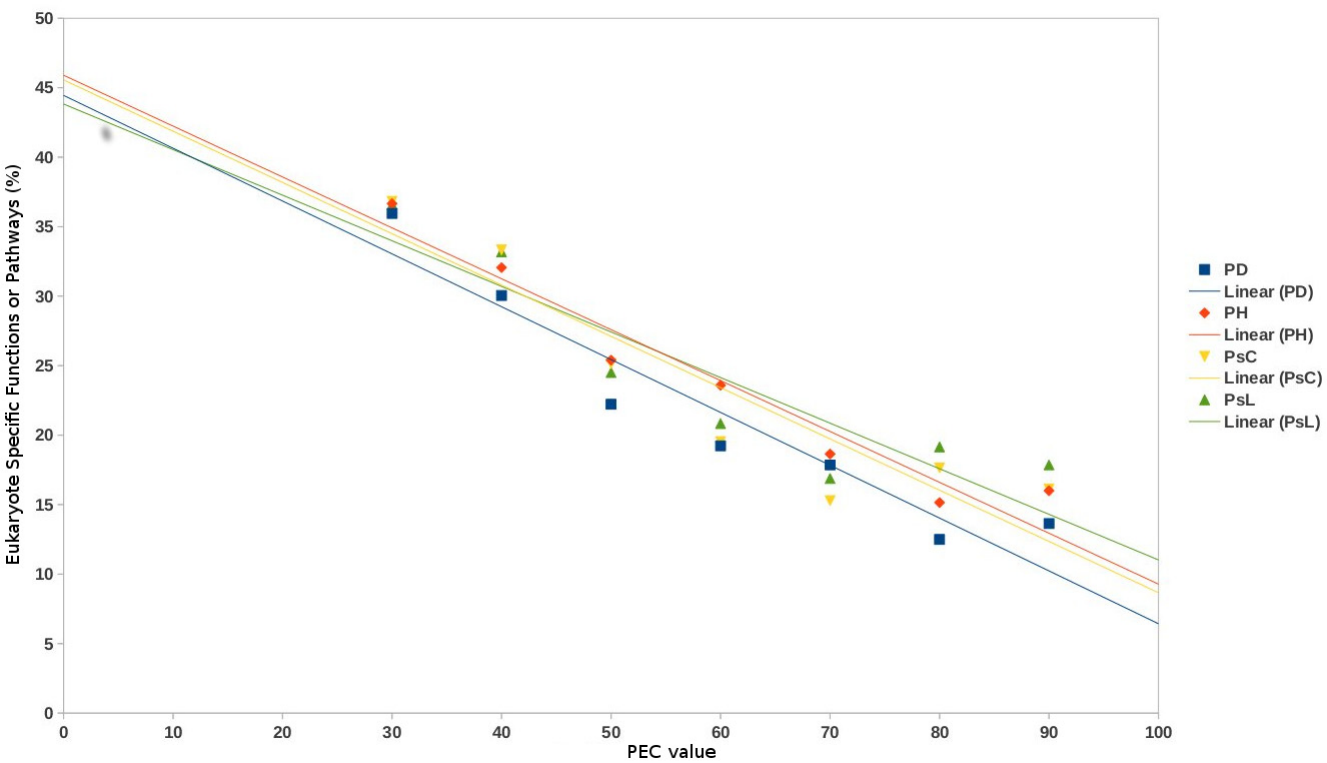
Validation of Pathway Exclusion Cut-off (PEC) value. Graphical depiction of the quantum of eukaryotic functions predicted by Vikodak at different PEC values. Results indicate a consistent decrease in the proportion of predicted eukaryotic functions with increasing PEC value. While PD and PH represent datasets comprised of microbiome samples obtained from ‘subjects with Periodontitis’ and ‘Periodontally healthy controls’ (Griffen *et al*., 2012), PsC and PsL represent samples pertaining to Psoriasis Control and Psoriasis Lesional datasets (Alekseyenko *et al*., 2013).

Vikodak's 'Pathway Exclusion Cut-off (PEC)' parameter is expected to have utility in removing the subset of functions/ pathways (from the final predicted list) which were included based on the mere presence of a small proportion of genes/ enzymes corresponding to that function/ pathway. To evaluate the efficiency of this parameter, publicly available oral and skin metagenomic datasets [16,17] were processed using Global Mapper module of Vikodak at various PEC values (30–90). Results obtained at various PEC values were analysed with respect to the number of reported pathways, as well as, the reduction in the number of eukaryotic pathway predictions. Fig 4 graphically depicts the quantum of eukaryotic functions predicted by Vikodak at different PEC values. Results indicate a consistent decrease in the proportion of predicted eukaryotic functions with increasing PEC value. This utility in Vikodak is expected to be useful especially while analysing 16S datasets obtained from prokaryotic microbial communities (wherein the number of eukaryotic functions is expected to be minimal/ absent).

### Case Study highlighting biological relevance of Vikodak's functional predictions

91 publicly available 16S metagenomic datasets [17] corresponding to 32 sub-gingival samples from 30 Periodontally healthy control subjects (PH) and sub-gingival samples from 30 shallow (PS) and 29 deep (PD) periodontal pockets of subjects with Periodontitis were used as a case-study for highlighting the biological relevance of Vikodak's functional predictions. These datasets were processed using all three modules of Vikodak and results were analysed for functional significance.

### (A) Analysis using Global Mapper

**Fig 5:**
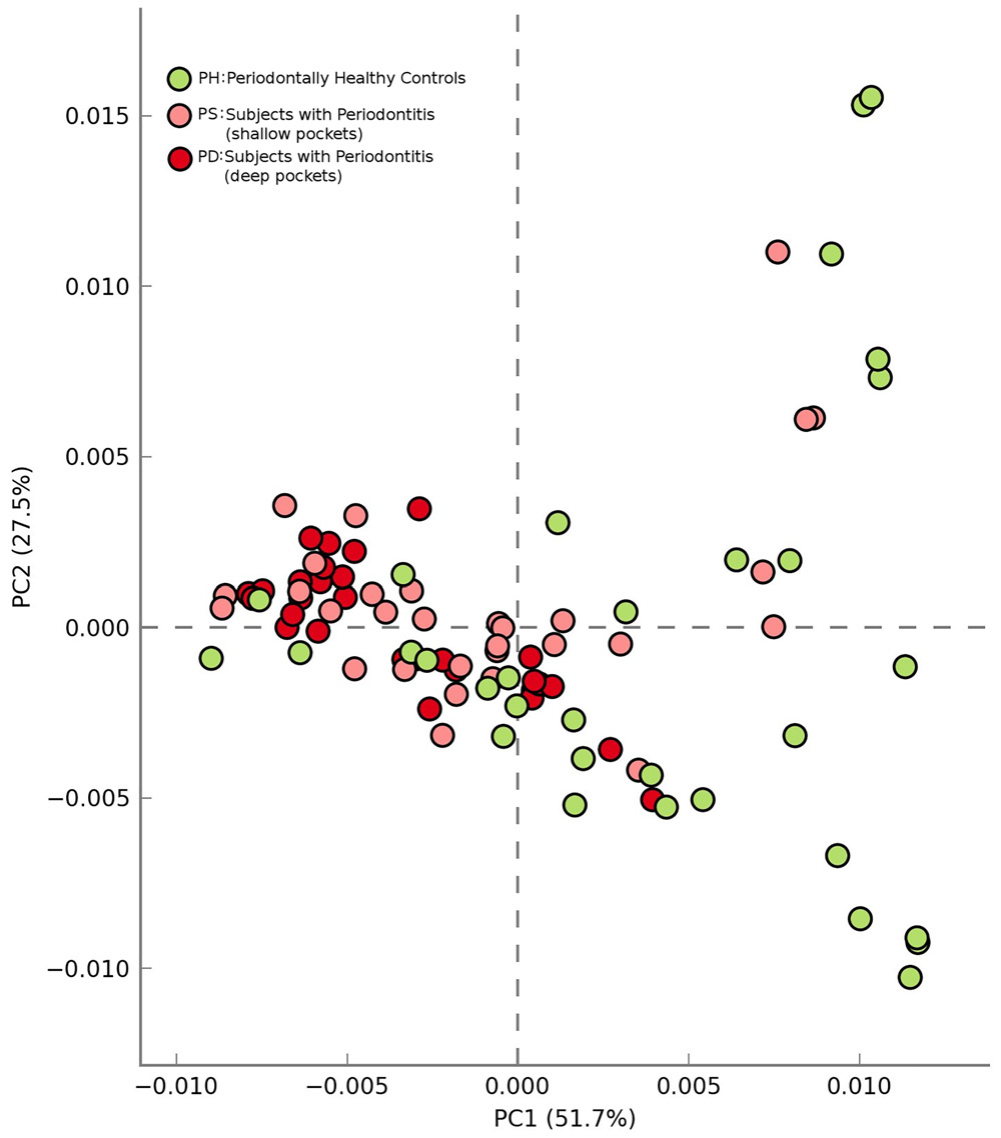
Ordination analysis of datasets pertaining to subjects with Periodontitis (PD, PS)and Periodontally healthy controls (PH) datasets using Vikodak derived functional profiles. PCA-based ordination analysis carried out on Pathway Abundance Profiles (obtained using Global Mapper module) of all 91 samples of Periodontitis datasets (Griffen et al., 2012). Cluster points in red and peach pertain to PD and PS datasets. Cluster points in green represent PH datasets.

Results of PCA-based ordination analysis (using STAMP v.2.0.8 [19]) carried out on ‘Pathway Abundance Profiles’ (obtained using Global Mapper module) of all 91 samples are depicted in Fig 5. Results indicate clustering of pathway abundance profiles belonging to shallow pocket (PS) and deep pocket (PD) samples from subjects with Periodontitis. In contrast, profiles obtained from datasets corresponding to Periodontally healthy controls (PH) were observed to have a spatially scattered pattern. Given that diseased samples have a distinct physiological profile (due to a cause or as a consequence) they are expected to manifest a relatively homogeneous signature in their microbiome profiles. The clustering pattern obtained using Vikodak are in sync with this assertion and results therefore seem to be relevant from a biological standpoint.

### (B) Analysis using ISFA and Local Mapper

**Fig 6:**
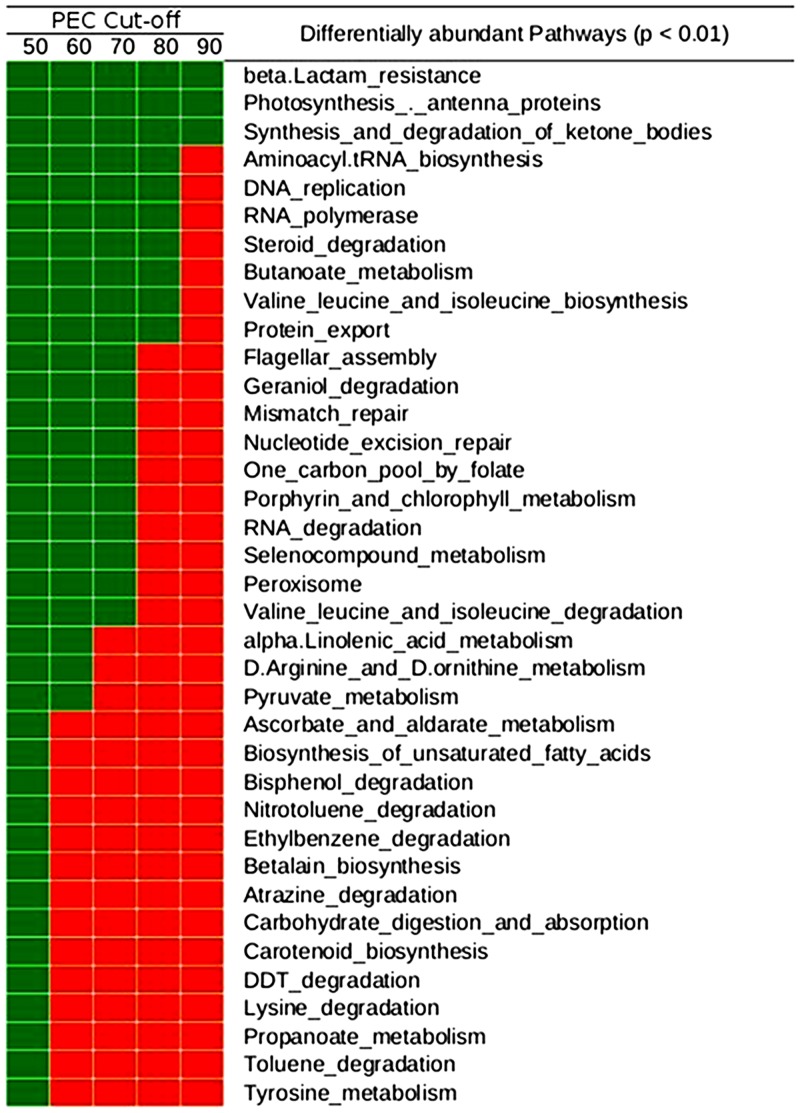
Results of ISFA pertaining to PD (subjects with Periodontitis, deep pockets) and PH (Peridontically healthy controls) datasets. Results of Inter Sample Feature Analysis (at various PEC values: 50–90) aimed at identification of significantly differentiating functions between PD and PH datasets (Griffen et al., 2012). Green color indicates that the corresponding feature was observed to be significantly differentiating under the given PEC value, while red color indicates the contrary. Identifying functions that are reported as significantly different at most PEC thresholds greatly increase the confidence in the set of functions identified as significantly different.

The Pathway Abundance Profiles (obtained at PEC values 30–90) of datasets corresponding to PD (subjects with Periodontitis, deep pocket) and Periodontally healthy control (PH) datasets were provided as input to the ISFA module (random sample size of 70%, p-value<0.01, number of iterations 1000, boot-strap threshold 70%). The objective was to highlight the utility of Vikodak in enabling automated identification of the subset of functional features that are significantly different between the compared datasets. Fig 6 summarizes the results of this analysis.

'beta-lactam resistance', 'photosynthesis (antenna proteins)' and 'synthesis and degradation of ketone bodies', functions known to play key roles in progression of Periodontitis [20–22], are observed to be significantly differentiating between PD and PH datasets at all PEC values. Given that the occurrence of beta-lactamase positive bacterial species in subjects affected with periodontitis/ gingivitis is well documented in literature [20,23,24], results obtained with the 'Independent Contribution' sub-module of Global Mapper were further analysed to obtain additional insights regarding the contributions of individual microbes (in both PD and PH) to beta-lactam resistance. This analysis indicates higher levels of beta-lactam resistance function in all those bacterial genera that have been previously associated with periodontitis (Table 1).

**Table 1:** beta-lactam resistance contribution in datasets obtained from Periodontally healthy controls(PH) and subjects with Periodontitis (PD). Individual contributions of resident microbes of PH (healthy samples) and PD (deep pocket samples) of Periodontitis study (Griffen *et al*., 2012) towards 'beta-lactam resistance' function. Generahighlighted in 'bold font' pertain to taxa that have been previously associated with periodontitis.

Genera	beta-lactam resistance (% contribution)	
	PH	PD
***Veillonella***	2.00	2.50
*Brachybacterium*	0.30	0.00
*Campylobacter*	0.80	1.70
*Capnocytophaga*	0.90	1.00
*Catonella*	0.00	0.30
*Comamonas*	0.10	0.00
*Acinetobacter*	1.00	0.40
*Corynebacterium*	0.40	0.30
*Actinomyces*	0.60	0.30
***Dialister***	0.00	0.10
*Eubacterium*	0.10	1.20
***Fusobacterium***	19.80	47.30
*Gemella*	0.20	0.10
*Haemophilus*	0.10	0.00
*Neisseria*	0.40	0.20
***Porphyromonas***	0.10	2.60
***Prevotella***	1.50	10.50
***Selenomonas***	0.20	2.30
*Arthrobacter*	0.70	0.00
***Streptococcus***	70.60	28.60
*Mycoplasma*	0.00	0.10
***Peptostreptococcus***	0.00	0.10
***Tannerella***	0.00	0.50

**Fig 7:**
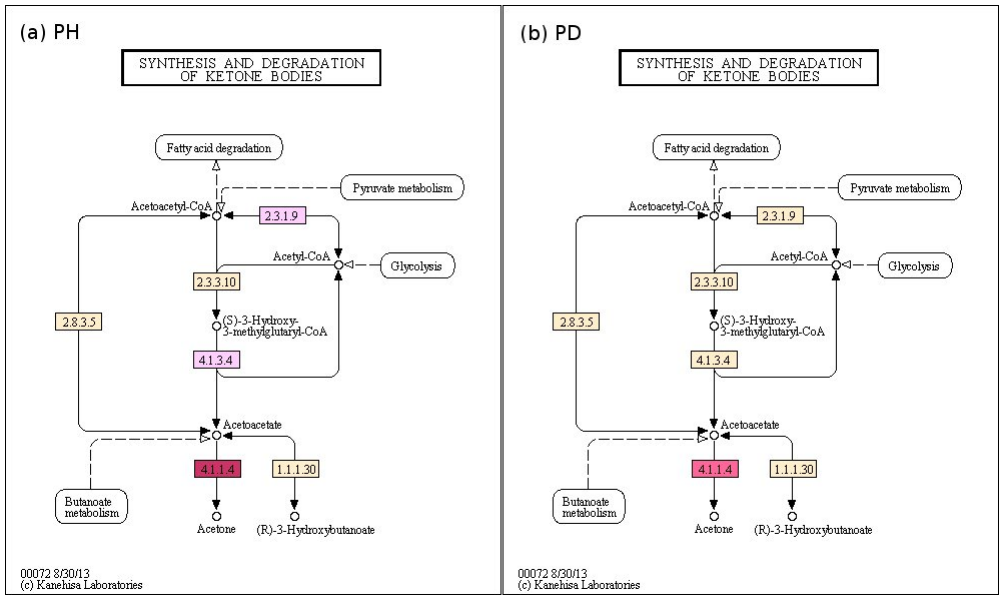
Utility of ‘User-Data-Mapping-File’ in comparing PD (subjects with Periodontitis, deep pockets) and PH (Periodontally healthy controls) datasets using Local Mapper. Graphical visualization of (qualitative) relative abundance of various enzymes pertaining to 'Synthesis and Degradation of Ketone Bodies' function in PH and PD datasets (Griffen et al., 2012). Higher the intensity of color, greater is the relative abundance of the corresponding enzyme. The visualization was created using the 'User-Data-Mapping-File (example: S2 File, section J)' generated using Local Mapper Module of Vikodak.

**Fig 8:**
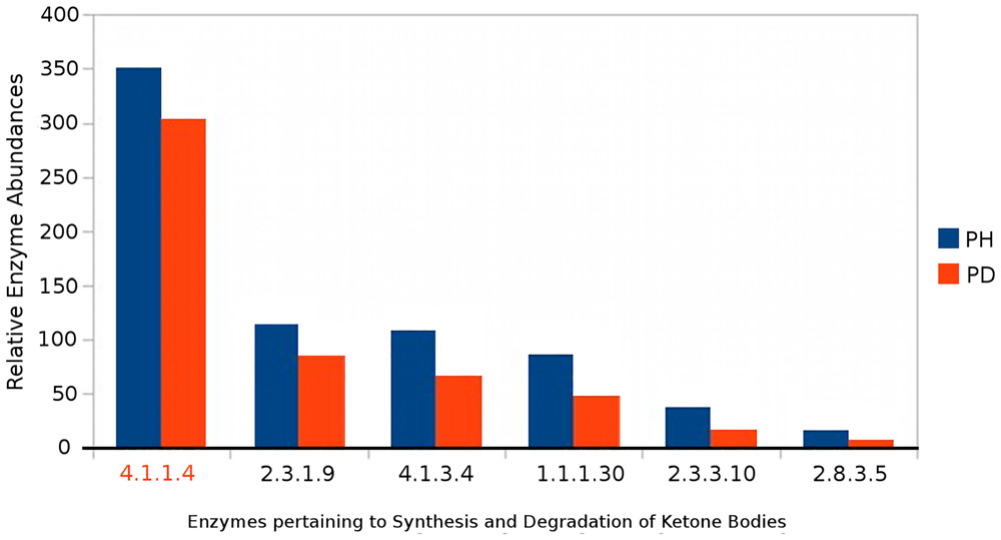
Comparison of PD (subjects with Periodontitis, deep pockets) and PH (Periodontally healthy controls) datasets in terms of 'Synthesis and Degradation of Ketone Bodies' function using Local Mapper. Graphical visualization of (quantitative) relative abundance of various enzymes pertaining to 'Synthesis and Degradation of Ketone Bodies' function in PH and PD datasets (Griffen et al., 2012). Highlighted EC number corresponds to ‘acetoacetate decarboxylase’ that catalyzes the degradation of acetoacetate (ketone body). The visualization was created using the 'Effective-Enzyme-Abundance-Profiles (example: S2 File, section J) generated using Local Mapper Module of Vikodak.

Similarly, ketoacidosis (i.e. presence of ketone bodies in breath) has been recently suggested as a causative factor for halitosis (malodour) in diseases like periodontitis [22]. Probing the enzymes profiles related to the function—'synthesis and degradation of ketone bodies' (using Vikodak's 'Local Mapper' module) in datasets from Periodontally healthy controls (PH) and subjects with Periodontitis (PD) indicate an overall higher metabolism of ketone bodies in PH datasets as compared to PD datasets (Figs 7 and 8). Furthermore, functions (enzymes) corresponding to degradation of ketone bodies are found to exhibit effectively lower abundance in diseased samples, as compared to the healthy samples (Figs 7 and 8). Thus, results obtained using various modules of Vikodak appear to indicate a significant role of oral microbes in periodontitis associated halitosis. Table 2 provides a (literature-mined) [25–29]summary of the biological relevance of other features identified (by Vikodak) as significantly differentiating between PD and PH datasets.

**Table 2:** Relevance of Vikodak derived differentiating (functional) features between PD and PH datasets. A (literature-mined) summary of the biological relevance of other features identified (by Vikodak) as significantly differentiating between datasets obtained from Periodontally healthy controls (PH) and subjects with Periodontitis (PD). These datasets were obtained from Griffen et al. (2012).

Pathway	Pathway Abundance*	Report(s) in literature and discussion w.r.t Vikodak	Reference
Steroid Degradation	Low	Salivary concentrations of sex steroid hormones have been observed to be high in Periodontitis patients. It was suggested that there exists a co-relation between altered production of sex steroid hormones and periodontitis. We hereby suggest that the increased salivary concentrations of the steroid hormones in Periodontitis patients may be attributed to the decrease in bacterial steroid degradation function.	Vittek et al., 1984 [26]
beta-Lactam resistance	High	Occurrence of beta-lactamase positive bacterial species in subjects affected with periodontitis/gingivitis has been reported in numerous scientific reports. In the present study, beta-lactam resistance function was observed to exhibit an increasing trend in all those bacterial genera that have been previously associated with periodontitis. This analysis was possible due to 'independent contributions' algorithm of Vikodak's Global Mapper.	Rams et al., 2013 [20]; Herrera et al., 2000 [24]; Kesic et al., 2008 [25]
Photosynthesis (antenna proteins)	Low	Tissue damage due to higher prevalence of reactive oxygen species in Periodontitis niche has been reported in previous scientific research. ROS are generated due to errors in oxidative phosphorylation leading to inappropriate reduction of terminal oxygen in ETC; Photosynthesis (antenna proteins) function corresponds to genes encoding bacterial oxidative phosphorylation. It was observed through Vikodak's ISFA that bacterial oxidative phosphorylation (which is similar to mitochondrial Oxidative phosphorylation) has higher effective abundance in healthy subjects as compared to the periodontitis affected subjects. It is thus suggested that the oral microbiota of healthy subjects supplements the mitochondrial ETC in efficient reduction of oxygen to water, and helps in fighting oxidative stress more effectively than that of the periodontitis subjects, which might lead to further aggravation of the periodontitis related tissue damage.	Dhotre et al., 2012 [21]
Synthesis and degradation of ketone bodies	Synthesis: High	Ketoacidosis (presence of ketone bodies) of breathe has been suggested as a causative factor for halitosis (malodor) in diseases like periodontitis in previous reports; Current observation of results obtained from Inter Sample Function analysis (coupled with analysis using Local Mapper) of periodontally healthy and diseased subjects indicated higher abundance of bacteria mediated 'synthesis of ketone bodies' function in diseased subjects as compared to the healthy ones. Also, functions corresponding to degradation of ketone bodies exhibited lower effective abundance in diseased samples, as compared to the healthy samples. It may thus be hypothesized that oral microbiota plays a significant role in periodontitis associated halitosis.	Eid H, 2014 [22]
	Degradation: Low		
Butanoate Metabolism	Acetate/Butyrate: High	Butyric acid and acetate has been known to effect development of periodontitis via oxidative stress generation and induction of apoptosis. Recent reports have also described the high prevalence of acetate/butanoate ratio in periodontitis associated biofilms; Butanoate metabolism function directed towards acetate/butyrate synthesis was found to have high effective abundance in periodontally diseased subjects as compared to the healthy subjects through ISFA and Local Mapper of Vikodak. Enzymes catalyzing Acetoin synthesis (an important bacterial metabolite for controlling acidification) were also observed to be upregulated in microbiota of healthy subjects. It may thus be suggested that higher production of butanoate metabolites like acetate and butyrate by the periodontopathic bacteria lead to aggravation of inflammatory and stressful conditions in the diseased tissue. Also, down regulation of acetoin synthesis might lead to lack of control over acidification by the oral microbiota.	Chew et al., 2012 [27]; Lu et al., 2014 [28]; Ochiai et al., 2011 [29]
	Acetoin: Low		

*Pathway Abundance: Relative abundance of given pathway in PH datasets as compared to PD datasets.

### (C) Function-driven Microbial Interaction Networks

Individual contributions of various microbes towards different functions (at all KEGG hierarchy levels, viz. Pathways, class, and super-class) obtained using Vikodak can, in principle, be used for generating a microbial correlation network. Analysis of such a network (in terms of various network properties viz. Degree, Betweenness, Network Density, etc.) is expected to facilitate the identification of (1) microbes that play key functional roles in a given community, and (2) microbes (or group of microbes) that are functionally synchronised/ antagonistic with respect to each other. Furthermore, comparing such networks generated for two communities (e.g. healthy vs. diseased states) enables not only identification of changes in key regulatory members, but also helps in tracking the transition in overall functional interactions between the resident microbes. It is pertinent to note here that the networks discussed above are unique with respect to characterising functional interactions at a community level (as opposed to a taxonomic abundance derived network). Results obtained from the 'independent contributions' sub-module of Global Mapper (in Vikodak) enable generation of such function-based microbial networks, the latter utility being unavailable in any of the existing function prediction tools. S2 File (section E) represents the typical format of ‘pathway level functional profile of individual microbes’ generated by 'independent contributions' sub-module of Global Mapper. This profile is further used to infer function based inter-microbial interactions.

**Fig 9:**
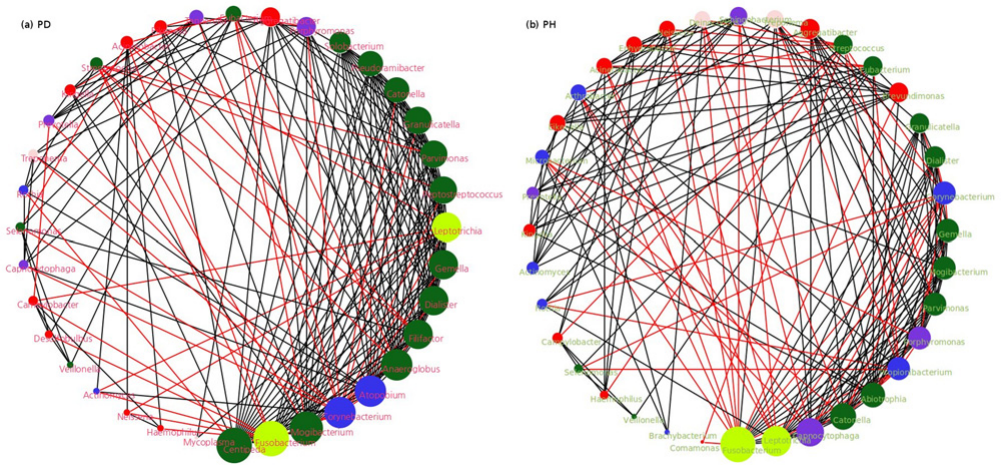
Function based microbial interaction network in (A) subjects with Periodontitis, deep pocket samples [PD] and (B) Periodontally healthy controls [PH]. Microbial Interaction Networks generated using the abundance data pertaining to the functional contribution of various microbes in (A) PD datasets (B) PH datasets (Griffen et al., 2012). The networks may thus be interpreted as an index of functional interactions/dynamics between the resident microbes of PD/PH environment. The networks are plotted in 'Degree Sorted Circular Layout' using Cytoscape 3.0.2. Bigger nodes pertain to microbes with 'higher degree' of interaction, while smaller nodes indicate the microbes with 'less degree' of interaction. Color of the edges serve as an index of positive (black) and negative (red) interactions between the constituent microbes of the networks. Color of the nodes pertain to different phylum affiliations of the microbes (red: Proteobacteria; blue: Actinobacteria; green: Firmicutes; violet: Bacteroidetes; fluorescent green: Fusobacteria; peach: Other).

Fig 9 depicts function-based microbial networks generated using the pathway level functional profile of individual microbes of PD and PH datasets, obtained using ‘Independent contributions’ sub-module of Vikodak. The networks in the figures were generated using Cytoscape 3.0.3 [30]. The methodology used for generating these networks is similar to that used in earlier studies [31]. Centipeda, a genus reported to have significant association with periodontitis [32,33], is observed to be the highest degree node in the PD network. Interestingly, this genus is found to be absent in the network generated using PH datasets. In addition, a noticeable decrease in number of negative interactions (S6 Fig) between the resident microbes of the PD environment is observed. This indicates synchronization of functional interactions between microbes in the diseased environment. This synchronisation is possibly due to specific physiologic condition(s) in the diseased state. Given this condition, microbes possessing (common) functional capabilities to inhabit that niche are expected to have a higher number of positive interactions as compared to the PH datasets.

## Discussion

**Fig 10:**
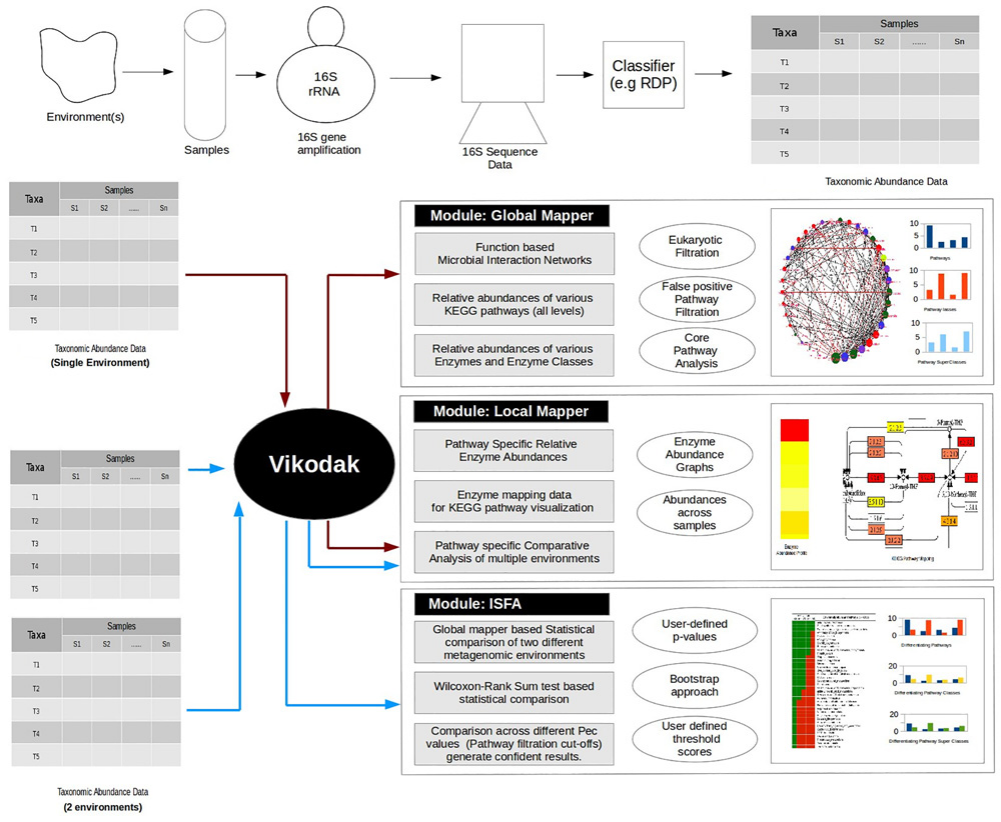
Schematic summary of Vikodak. A schematic depiction of the overall workflow and application(s) of Vikodak. Three distinct functional modules, viz. Global Mapper, Inter Sample Feature Analyzer (ISFA), and Local Mapper, each catering to specific end-user requirements are depicted. Given a microbial abundance data profile of an environmental sample (e.g. 16S sequencing data classified using RDP classifier), the Global Mapper module enables (a) an *in silico* estimation of the relative abundance of various metabolic pathways in that sample, (b) quantifying the contribution of individual microbes (in that sample) to the predicted functions (at all three tiers of KEGG hierarchy), and (c) identification of the core set of metabolic functions defining a particular environment. The ISFA module in Vikodak is an extension of the Global Mapper module and is designed for performing a rigorous (pair-wise) comparative statistical analysis of the (inferred/predicted) functional profiles generated from two or more environments. The Local Mapper module further enables end-users to probe, in greater detail, the enzyme abundance profile(s) of individual metabolic pathway(s) identified as (a) the 'core' in one or more environments, or (b) differentially abundant between two or more environments.

In addition to providing information about the presence/ abundance of various genes/ proteins/ metabolic-pathways encoded by various microbes inhabiting a given environment, an ideal function characterization tool should be able to provide insights pertaining to the functional interactions between the resident bacteria. A comparison of such information generated from two or more environments is expected to provide insights relevant from a biological standpoint. Vikodak—the tool presented in this study attempts to automate the overall process of obtaining and/ or comparing the functional profiles(s) of given environment(s). Fig 10 schematically depicts various modules and associated utilities of Vikodak.

In comparison to existing functional annotation tools, the functional profiles obtained using the 'Global Mapper' module of Vikodak contain detailed information with respect to the core set of functions as well as the contribution of individual microbes to various functions identified in the studied environment(s). This facilitates end-users to decipher functional relationships between various resident microbes. Furthermore, the 'ISFA module' of Vikodak automates the functional comparison between a pair of environments. Given that the comparison work-flow employs a boot-strap approach (and also provides end-users the option to choose appropriate statistical thresholds), Vikodak represents a reliable one-stop package that infers as well as compares (in rigorous statistical terms) the functional profiles of the studied environment(s). The 'Local Mapper' module further complements the functionalities of the other two modules by enabling an in-depth (enzyme-level) analysis of specific metabolic pathways that are of interest to the end-user.

In ideal circumstances, the process of inferring the presence of a pathway in an environmental sample should necessarily take into consideration the occurrence of a minimum quorum of genes/ enzymes encoding the pathway. For instance, it is inappropriate to report the 'presence' of a pathway (constituted of 20 enzymes) based on finding just 1 or 2 enzymes in the studied environment. The PEC value parameter in Vikodak duly takes care of this requirement, thereby enabling end-users to focus only on results which are bereft of possible false positive predictions. Comparison of microbial communities from two environments (using Vikodak's ISFA module) and identifying functions that are reported as significantly different at most PEC thresholds (Fig 6) greatly increases the confidence in the set of functions identified as significantly different.

On a conceptual note, Vikodak considers enzymes as the basic functional units. This in turn has enabled the development of a relatively simplistic (yet effective) modules catering to various aspects of function prediction. The back-end data in Vikodak is therefore based on a compilation of EC copy numbers (from greater than 37000 bacterial genomes) sourced from two major data repositories viz. PATRIC and IMG [8, 9]. Given that current taxonomic classification algorithms [34–39] provide assignments at various levels of taxonomic hierarchy, the consensus mapping data in Vikodak has therefore been computed at all taxonomic levels. Vikodak is enabled to process taxonomic abundance data (generated using any of the currently available classification tools). With its additional provisions for comprehensive functional analysis/ comparison, deeper insights and more flexibility at the user end, Vikodak is expected to add value to the family of currently available tools for 16S based function prediction.

## Supporting Information

S1 Fig EC copy number z-score divergence profile. Dough nut plots depicting the proportions of various EC copy number z-score ranges observed at various taxonomic levels.
(TIFF)

S2 Fig KEGG hierarchy and Vikodak’s back-end hashmaps. A schematic representation of the three levels of KEGG pathway hierarchy and corresponding hash-maps used in the back-end datasbase of Vikodak.
(TIFF)

S3 Fig Underlying assumptions of Global Mapper’s algorithms. A schematic description of the assumptions underlying the two sub-modules/algorithms of Global Mapper module of Vikodak. Ep_i_ refers to the Enzyme pool contributed by the i^th^ bacterium (and so on). P_i_1 refers to the abundance of Pathway (P1) contributed by the i^th^ bacterium using its enzyme pool (Ep_i_).
(TIFF)

S4 Fig Schematic representation of Global Mapper workflow. A schematic representation of the work-flow employed in Global Mapper module of Vikodak and the format of various outputs generated using this module. Various types of analyses that can be performed using the generated outputs are also indicated.
(TIFF)

S5 Fig Illustration of ‘Graphic visulaization’ of a KEGG pathway using ‘user data mapping’ generated by Local Mapper module of Vikodak. Visual depiction of the relative adbundance levels of various enzymes belonging to ‘Glycine, Serine and Threonine Metabolism’ KEGG pathway obtained using the ‘user data mapping’ file generated by Local Mapper module of Vikodak (please refer S2 File, section J, to access sample ‘user data mapping file’).
(TIFF)

S6 Fig Comparison of functional profile based microbial networks of datasets in terms of ‘nature of interaction’. Bar graphs representing the comparison of the quantum of negative interactions between the microbes residing in healthy and diseased environments. While PH and PD represent datasets pertaining to Periodontally healthy controls (PH) and subjects with Periodontitis (PD), PsC and PsL represent samples pertaining to Psoriasis Control and Psoriasis Lesional datasets.
(TIFF)

S1 File Detailed algorithms of Global Mapper module of Vikodak. Detailed algorithms of Global Mapper module of Vikodak, with description of the methodology followed in implementing various noise and error reduction provisions.
(PDF)

S2 File Sample files depicting input/ output formats for various modules of Vikodak. This file contains the snapshots of sample input files that can be used for various modules of Vikodak. The snapshots of various types of outputs/results generated by Vikodak are also included in this file.
(PDF)

S1 Table Summary of various datasets employed for validation of Vikodak. A tabulated summary of various datasets employed to validate the utility of various modules of Vikodak.
(DOCX)
