# Supplementary figures and images for: Vikodak - A Modular Framework for Inferring Functional Potential of Microbial Communities from 16S Metagenomic Datasets
Source: PLoS One. 2016 Feb 5;11(2):e0148347. doi: 10.1371/journal.pone.0148347 (PMC4746064; doi:10.1371/journal.pone.0148347)

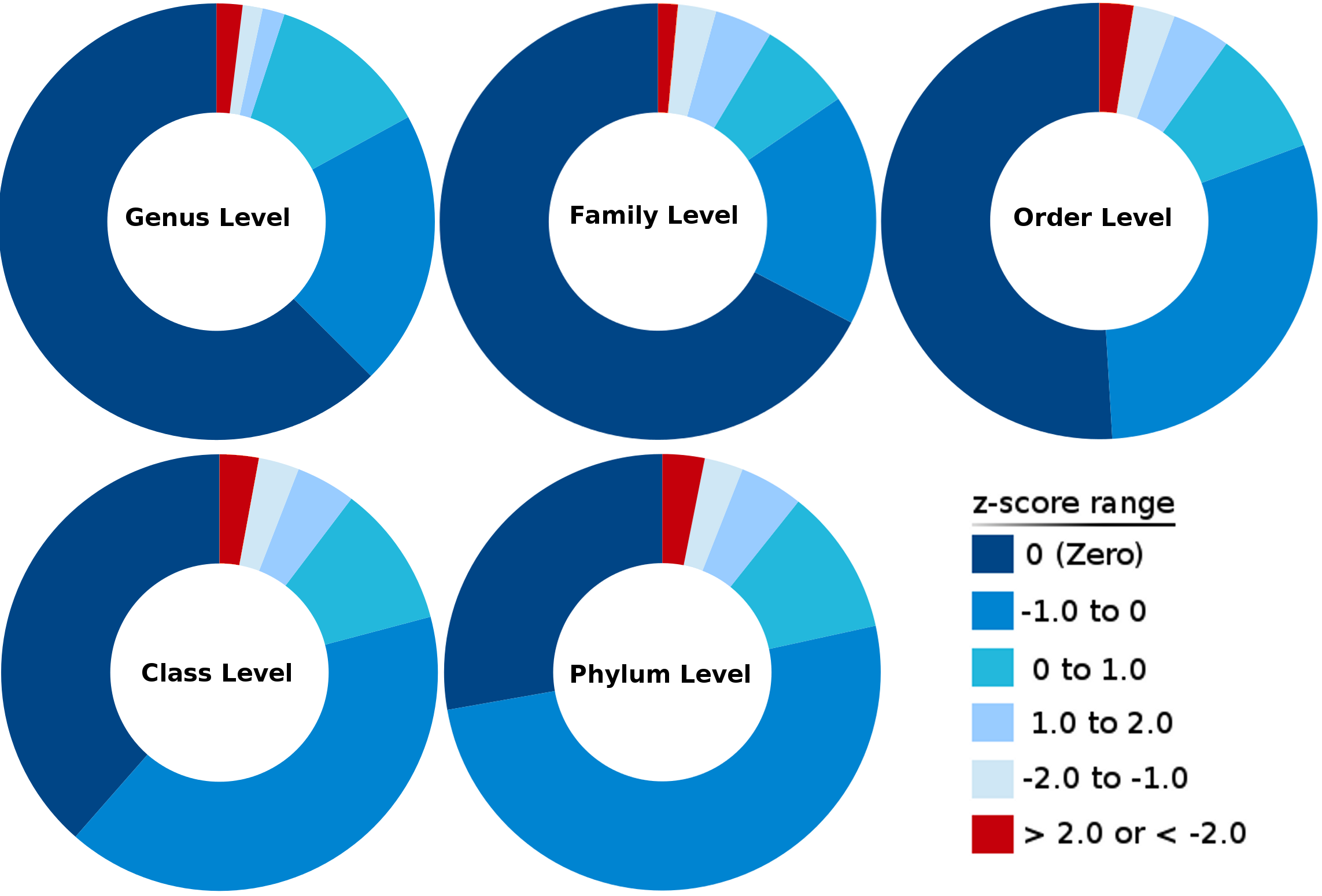

Supplement: S1 Fig — Dough nut plots depicting the proportions of various EC copy number z-score ranges observed at various taxonomic levels. (TIFF) [file pone.0148347.s001.tiff]

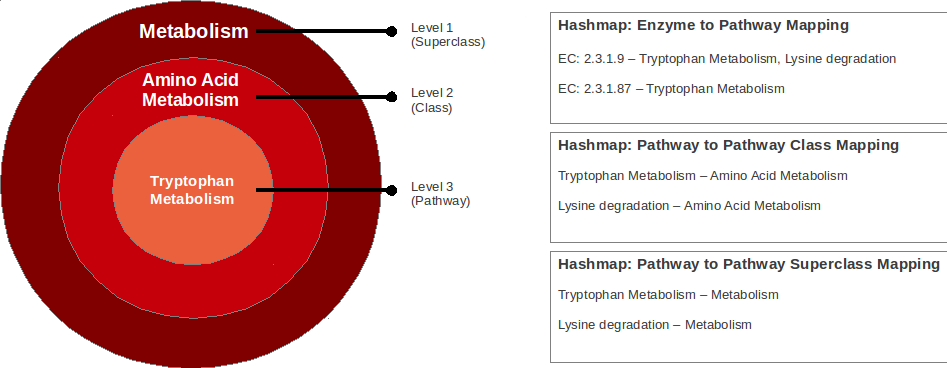

Supplement: S2 Fig — A schematic representation of the three levels of KEGG pathway hierarchy and corresponding hash-maps used in the back-end datasbase of Vikodak. (TIFF) [file pone.0148347.s002.tiff]

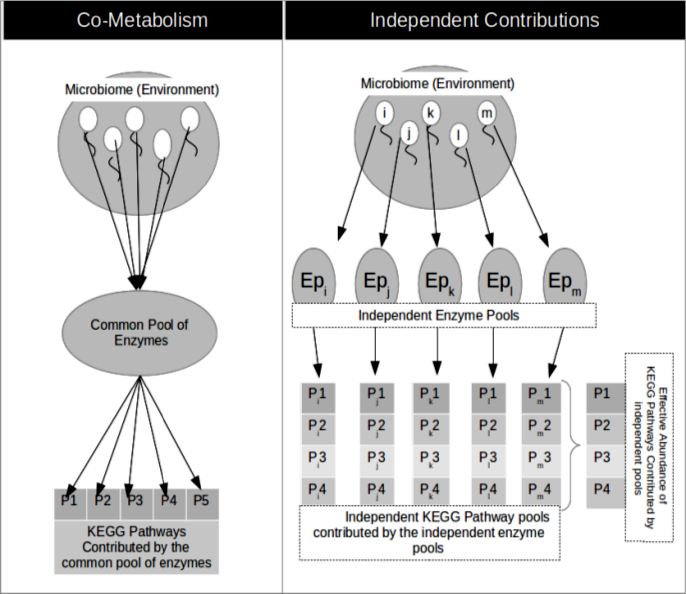

Supplement: S3 Fig — A schematic description of the assumptions underlying the two sub-modules/algorithms of Global Mapper module of Vikodak. Epi refers to the Enzyme pool contributed by the ith bacterium (and so on). Pi1 refers to the abundance of Pathway (P1) contributed by the ith bacterium using its enzyme pool (Epi). (TIFF) [file pone.0148347.s003.tiff]

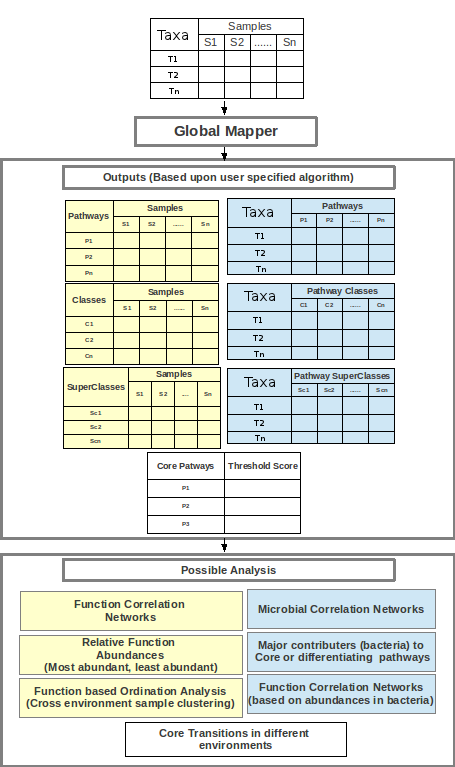

Supplement: S4 Fig — A schematic representation of the work-flow employed in Global Mapper module of Vikodak and the format of various outputs generated using this module. Various types of analyses that can be performed using the generated outputs are also indicated. (TIFF) [file pone.0148347.s004.tiff]

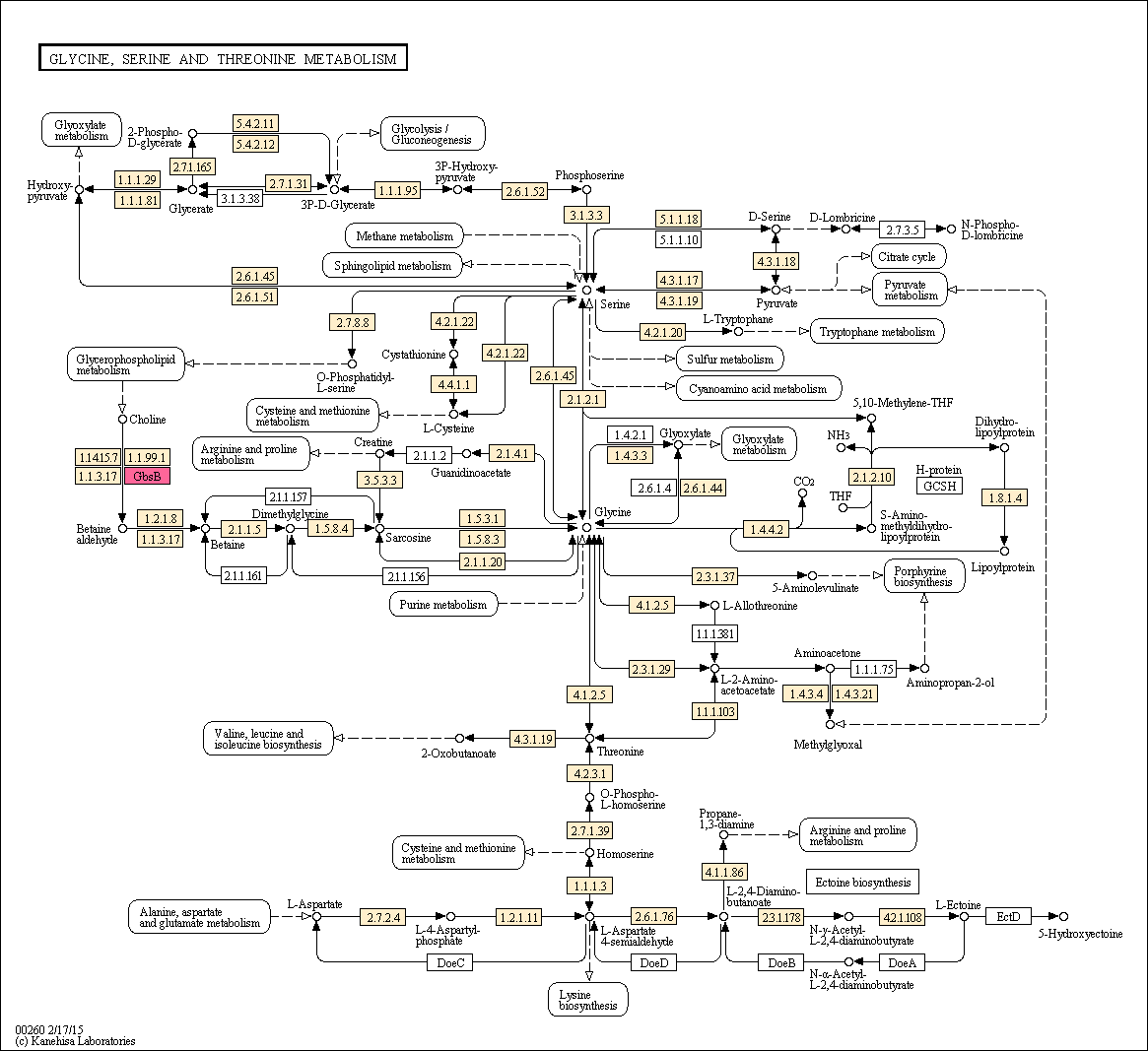

Supplement: S5 Fig — Visual depiction of the relative adbundance levels of various enzymes belonging to ‘Glycine, Serine and Threonine Metabolism’ KEGG pathway obtained using the ‘user data mapping’ file generated by Local Mapper module of Vikodak (please refer S2 File, section J, to access sample ‘user data mapping file’). (TIFF) [file pone.0148347.s005.tiff]

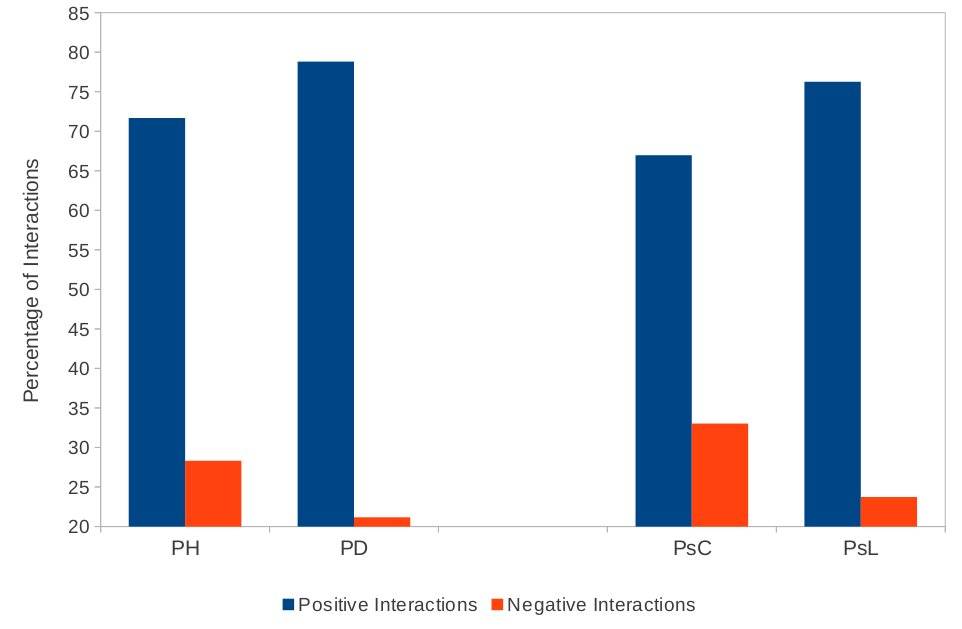

Supplement: S6 Fig — Bar graphs representing the comparison of the quantum of negative interactions between the microbes residing in healthy and diseased environments. While PH and PD represent datasets pertaining to Periodontally healthy controls (PH) and subjects with Periodontitis (PD), PsC and PsL represent samples pertaining to Psoriasis Control and Psoriasis Lesional datasets. (TIFF) [file pone.0148347.s006.tiff]
