# Supplementary material for: Vikodak - A Modular Framework for Inferring Functional Potential of Microbial Communities from 16S Metagenomic Datasets
Source: PLoS One. 2016 Feb 5;11(2):e0148347. doi: 10.1371/journal.pone.0148347 (PMC4746064; doi:10.1371/journal.pone.0148347)
Supplement: S1 File — Detailed algorithms of Global Mapper module of Vikodak, with description of the methodology followed in implementing various noise and error reduction provisions. (PDF) [file pone.0148347.s007.pdf]

Supplementary File 1: Detailed algorithms of Global Mapper module of Vikodak

Detailed algorithms of Global Mapper module of Vikodak, with description of the methodology followed in implementing various noise and error reduction provisions**.**

Section A: Global Mapper Work-flow

Both sub-modules of Global Mapper, i.e. *Co-metabolism* and *Independent contributions,* take as input, (16S amplicon sequencing derived) raw taxonomic abundance data (in tab-delimited format) corresponding to various microbes in the studied environmental sample(s). For ease of understanding, a sample input file is provided in Supplementary File 2 (section A). Both sub-modules of Global Mapper process this input file using the following sequence of steps –

STEP 1 - Normalization of Abundance Data

Abundance data normalization is done with respect to two aspects

a) Sample Size Normalization
b) 16S Copy Number Normalization

Sample size normalization (optional) ensures uniform scaling of the data, removing biases arising due to different sample sizes. For correcting the input abundance profiles for 16S copy number of various taxa, Vikodak uses a manually compiled database containing 16S copy numbers of microbial species). This database was compiled using information obtained from the RDP classifier distribution as well as from the ‘all.rnt' file available for download at the NCBI ftp site (ftp://ftp.ncbi.nih.gov/genomes/Bacteria/all.rnt.tar.gz).

STEP 2 - Generation of Enzyme Abundance Profile(s)

This stage computes the abundance profiles for enzymes present in various samples of the studied environment(s). Given the differences in the underlying assumptions, the two sub-modules of Global Mapper deduce these profiles differently as described below -

Co-Metabolism sub-module: Co-metabolism sub-module of Global Mapper assumes that microbes in an environment exist as a consortium and individual microbes contribute to a common enyzme pool. The microbial community represented by individual (normalized) abundance data profiles is therefore mapped against the pre-computed Vikodak's back-end database (please refer to main paper) to obtain enzyme profile(s) of each sample. This profile enlists all enzymes (along with their copy numbers) present in various taxa constituting the studied microbial community. In order to compute the effective abundance of individual enzymes in the studied microbial community, the enzyme copy number values of various taxa in individual samples are multiplied with the normalized abundance values of the corresponding taxa in respective samples. The following mathematical expression depicts the above computation -

$$E = \sum_{i=1}^{n} Ec_i \ x \ Ab_i$$

Wherein,

E = Effective Abundance Value of an Enzyme

Ec_i= Enzyme Copy Number value in i^{th}taxon of a sample

Ab_i= 16S and Sample Size Normalized Abundance Value (if chosen) of ith taxon in the given sample

n = Number of taxa expressing the Enzyme in the sample

Supplementary File 2 (section B) depicts the format of an 'Enzyme Abundance Profile' generated at the end of this step. It may be noted that the co-metabolism sub-module further processes information present in the Enzyme Abundance Profile to compute (and provide) information pertaining to the abundances of six enzyme classes (viz. oxido-reductases, ligases, lyases, hydrolases, tranferases, and isomerases) present in the studied environment.

Independent contributions sub-module: Independent contributions sub-module assumes that each microbe in a community has its own independent pool of enzymes with no co-metabolic associations between various microbes. Consequently, in contrast to generation of a single 'Enzyme Abundance Profile' for a given environment (as in the co-metabolism sub-module of Global Mapper), independent Enzyme Abundance Profiles are generated for individual taxa present in each individual sample of an environment. Therefore, S number of matrices are generated (where S is the number of samples in the microbial abundance data submitted as the input), each representing the individual Enzyme Abundance Data for various microbes of a sample.

STEP 3 - Computation of Metabolic Pathways

The objective of this step is to use Enzyme Abundance Profile(s) for computing the relative abundance of metabolic pathways at all three levels of KEGG hierarchy. The methodology followed by the two sub-modules of Global Mapper during this computation is described below -

Co-Metabolism sub-module: The union of various enzymes (EC numbers) present in the Enzyme Abundance Profiles of all samples (in the studied environment) is mapped against the pre-computed 'Enzyme to KEGG Pathway hashmap (please refer to Supplementary Figure 2**)** for tagging each EC number (enzyme) of the Enzyme Abundance Profile(s) to different KEGG pathways. Post tagging, Pathway Abundance Profile(s) for each individual sample is calculated using the following mathematical expression -

$$P = \sum_{i=1}^{n} E_i$$

Wherein,

P = Effective Abundance Value of a KEGG Pathway in a sample

E_i= Effective abundance of ith enzyme involved in the Pathway in the Enzyme Abundance data for the sample

n = Number of enzymes present in the sample corresponding to the KEGG pathway

Supplementary File 2 (section C) represents an example of Pathway Abundance Profile.

The Pathway Abundance Profiles generated are subsequently used for computing Pathway Class and Super-Class Abundance Profiles (using respective hashmaps) and similar mathematical rules depicted below –

$$C = \sum_{i=1}^{n} P_i \quad | \quad Sc = \sum_{i=1}^{n} P_i$$

Wherein,

C = Effective Abundance Value of a KEGG Pathway class in a sample

Sc = Effective Abundance Value of a KEGG Pathway super-class in a sample

Pi= Effective abundance of ith Pathway under the Pathway class or Pathway super-class in the Pathway Abundance data for the sample

n = Number of Pathways present in the sample corresponding to the KEGG Pathway class or Pathway super-class

Supplementary File 2 (section D) represents the examples of Pathway Class and Super-class Abundance Profiles generated using Global Mapper.

Independent contributions sub-module: Similar to the co-metabolism sub-module, the independent contribution sub-module also aims at inferring the Pathway Abundance Profile(s) for the studied environment. However, the methodology involves computing the abundance of various KEGG pathways (and classes/ Superclasses) for each taxon of individual samples of the studied environment. Following mathematical expression is used for performing the aforesaid computation:

$$P_b = \sum_{i=1}^{n} E_i$$

Wherein,

P_b= Effective Abundance Value of the KEGG Pathway in b^{th} taxon of the sample

E_i= Effective abundance of ith enzyme involved in the Pathway, as obtained in the Enzyme Abundance data for the bth taxon

n = Number of enzymes (corresponding to the KEGG pathway) present in the taxon

Similarly, Pathway class and super-class abundances for individual taxa of each sample of the environment are computed (as mentioned earlier in the description for Co-metabolism module).

Once the functional profile for individual taxa of each sample is deduced, the computation of functional contribution of any microbial taxon in the overall environment can easily be performed using 'mean or median or relative contribution value' of the sample-wise functional contributions of the given taxon. It may be noted that, 'relative contribution' is calculated using the following mathematical expression:

$$Rc_f = \frac{\sum_{i=1}^{N} A_i}{\sum_{F=1}^{f} \sum_{i=1}^{N} A_i}$$

Wherein,

Rc_f = Relative contribution of fth feature, amongst total F features

A = Abundance value of the fth feature in i^{th} sample (total number of samples being N)

The features in the above expression refer to the row-names in inferred functions abundance data for individual samples, and may pertain to "Pathways/ Classes/ Superclasses"

The individual contribution(s) of various constituent taxa (in each sample) are used to infer Pathway Abundance Profiles for individual samples of the environment using the following mathematical expression –

$$P = \sum_{b=1}^{m} P_b \quad | \quad \text{For } P_b = \sum_{i=1}^{n} E_i$$

wherein,

P = Effective Abundance Value of a KEGG Pathway in a sample

P_b= Effective Abundance Value of the KEGG Pathway in b^{th} taxon of the sample

Ei= Effective abundance of ith enzyme involved in the Pathway in the Enzyme Abundance data for the bth taxon

m = Number of taxa mapped in the back-end database amongst those present in the input data

n = Number of enzymes present in the taxon corresponding to the KEGG pathway

Supplementary File 2 (section E) depicts the format of the Pathway Abundance Profile(s) of individual microbial taxa of an environment. The format of the Pathway Abundance Profile(s) for individual samples of an environment however remains identical to those indicated in Supplementary File 2 (section C).

Step 4 – Inferring Core Pathways

Both sub-modules (Co-metabolism and Independent contributions) of Global Mapper provide end-users an option for identifying 'core-pathways' present in a given environment. From the Pathway Abundance Profiles (at level 3 of the KEGG hierarchy), a bootstrapping mechanism is used for

identifying and reporting the subset of pathways having a minimum abundance of 0.1% in at least 80% of the samples as 'core-pathways'. The bootstrapping approach iteratively picks (at random) 70% of the samples and identifies core-pathways in that set of samples. This process is iterated 1000 times. Pathways reported as core in at least 70% of the iterations are tagged as 'core pathways' with a boot-strap score of 70 or above. Supplementary File 2 (section F) represents an example of a core-pathway result file generated using either of the two sub-modules of Global Mapper.

Section B: Noise and Error Reduction Provisions

Pathway Exclusion Cut-off (PEC): A major limitation of the current methods for function prediction is the inability to account for the constituents of a metabolic pathway. In other words, a metabolic pathway might be manifested by the joint expression of over 30 genes/ enzymes, but mere expression/ presence of 1-5 genes/ enzymes might not lead to the expression of the associated pathway. It is thus crucial to define a parameter for filtering pathways based on the proportion of various pathway associated genes/ enzymes expressed by the microbiota. Considering the need for such a parameter, Pathway Exclusion Cut-off (PEC) value has been defined in both algorithms of Global Mapper. PEC value is defined as the minimum percentage of genes/ enzymes belonging to any metabolic pathway that must be expressed by a given microbiota for tagging that pathway as being expressed by the microbiome. For example, a PEC value of 30 would mean that a given microbe/ microbiome must express at least 30% of genes/ enzymes belonging to any metabolic pathway for considering that pathway as being expressed. Both modules of Global Mapper have been developed in such a way that apart from providing the raw hits (where raw hits refer to the most inclusive criteria wherein even the expression of a single enzyme/ gene is considered for presence of a metabolic pathway) they provide the KEGG pathway (all three level) expression profiles for the microbial abundance data at various PEC values (30-90) as well.

Removal of Eukaryote-Specific Functions: A provision for removal of all those functions which are exclusive to eukaryotes (from the final functional inferences) has been provided to the users of Vikodak. A back-end data pertaining to the list of eukaryote-specific pathways was developed through literature and KEGG database curation for this purpose. The list includes functions/ KEGG pathways like MAPK signalling pathway, Oocyte meiosis, Plant hormone signal transduction, Rheumatoid arthritis, Vascular smooth muscle contraction, etc. Users have the choice to include/ exclude such functional hits in the final results.

Choice of Central tendencies (Mean, Median and Relative Contribution): Both sub-modules (Co-metabolism and Independent contributions) of Global Mapper offer options to users to analyse the function abundance data for various samples of an environment in terms of user defined central tendencies. A user can choose either Mean, Median or Relative contribution as the central tendency for analysing the effective abundances of various functions.

In addition, in case of the Independent Contributions algorithm, users are provided with three options (Mean, Median and Relative contribution) for executing this sub-module of Global Mapper. The options correspond to the decision for central tendency of the abundances of various functions contributed by each microbial taxon in the environment. This choice thus enables the computation of the representative functional abundances contributed by various resident microbes of the environment as a whole. Supplementary File 2 (section G) represents an example of the representative functional abundances contributed by various resident microbes of an environment.
