# Supplementary material for: Vikodak - A Modular Framework for Inferring Functional Potential of Microbial Communities from 16S Metagenomic Datasets
Source: PLoS One. 2016 Feb 5;11(2):e0148347. doi: 10.1371/journal.pone.0148347 (PMC4746064; doi:10.1371/journal.pone.0148347)
Supplement: S2 File — This file contains the snapshots of sample input files that can be used for various modules of Vikodak. The snapshots of various types of outputs/results generated by Vikodak are also included in this file. (PDF) [file pone.0148347.s008.pdf]

Supplementary File 2: Sample files depicting input/ output formats for various modules of Vikodak

This file contains the snapshots of sample input files that can be used for various modules of Vikodak. The snapshots of various types of outputs/results generated by Vikodak are also included in this file.

The page numbers may be clicked for direct reference to the listed sample file.

A. Input File for Global Mapper Module of Vikodak (**Page: →2←**)

B. 'Enzyme Abundance Profile' generated using Co-Metabolism sub-module of Global Mapper in Vikodak (**Page: →3←**)

C. ‘Pathway Abundance Profile’ generated using Global Mapper module of Vikodak (**Page: →4←**)

D. ‘Pathway class’ and ‘super-class’ files generated using the Global Mapper module of Vikodak (**Page: →5←**)

E. ‘Individual functional contributions’ file generated using Independent Contributions sub-module of Global Mapper (**Page: →6←**)

F. Sample ‘Core-Pathway’ results generated using Global Mapper module of Vikodak (**Page: →7←**)

G. Sample ‘representative functional abundance’ data generated by Global Mapper module of Vikodak (**Page: →8←**)

H. Sample Input and Output files of ISFA module of Vikodak (**Page: →9←**)

I. Sample input files for Local Mapper module of Vikodak (**Page: →10←**)

J. Sample output files generated by Local Mapper module of Vikodak (**Page: →11←**)

A. Sample Input File for Global Mapper module of Vikodak

Snapshot of the tab-delimited (16S amplicon sequencing derived) raw taxon-level abundance data file required as an input for both sub-modules of Global Mapper.

Features	Sample1	Sample2	Sample3	Sample4	Sample5	Sample6	Sample7	Sample8	Sample9	Sample10	Sample11
Parvimonas	94	320	43	373	17	123	253	2	20	2	350
Peptostreptococcaceae_incertae_sedis	66	1	0	65	0	0	0	0	15	0	17
Peptostreptococcus	7	0	0	35	0	0	0	0	0	0	169
Eubacterium	33	4	52	71	31	106	43	3	23	3	57
Johnsonella	1	2	1	0	0	0	0	0	6	0	6
Catonella	12	40	1	56	0	63	0	0	5	0	50
Clostridium_XIVa	1	0	0	0	0	0	0	0	0	0	0
Oribacterium	4	1	0	0	0	0	0	0	0	0	1
Clostridium_sensu_stricto	1	0	0	0	0	0	0	0	0	1	0
Mogibacterium	2	0	4	14	0	3	0	0	0	0	15
Selenomonas	50	13	47	41	29	145	9	2	1	4	6
Veillonella	126	122	341	103	29	181	237	165	1	170	5
Megasphaera	4	0	5	4	0	0	0	0	0	0	0
Centipeda	1	0	0	1	0	7	0	0	1	0	0
Dialister	14	1	12	28	1	11	12	0	3	1	78
Sporotalea	1	0	0	0	0	0	0	0	0	0	0
Streptococcus	520	3252	3080	864	1532	480	2490	3887	41	4740	105
Lactococcus	84	63	0	0	0	0	0	0	0	0	0
Enterococcus	245	35	0	0	0	0	0	0	0	0	0
Pilibacter	11	0	0	0	0	0	0	0	0	0	0
Granulicatella	54	78	575	111	327	37	70	70	31	100	2
Abiotrophia	2	0	14	1	67	5	0	3	0	4	0
Exiguobacterium	85	157	0	0	0	0	0	0	0	0	0
Gemella	33	53	209	206	16	39	0	404	7	502	55
Paenibacillus	5	0	0	0	0	0	0	0	0	0	0
Solobacterium	4	1	0	14	1	4	5	0	0	0	35
Rheinheimera	9	7	0	0	0	0	0	0	0	0	0
Pseudomonas	45	0	0	0	0	0	0	0	0	0	0

| Back to Page: →1← |

B. Sample 'Enzyme Abundance Profile' generated using Co-Metabolism sub-module of Vikodak

The format of an 'Enzyme Abundance Profile' generated using Co-Metabolism sub-module of Global Mapper. A tab-delimited file is generated, wherein first column represents various enzymes detected in the metagenomic environment and first row represents various samples of the environment (corresponding to the input file).

Features	Sample1	Sample2	Sample3	Sample4	Sample5	Sample6	Sample7	Sample8
1.10.2.2	20.492137	32.333171	62.026686	25.515052	54.407505	24.40045	45.527746	50.291773
1.10.2.-	3.0259647	2.509	0	0	0	0	0	0
1.10.3.11	0	0.00225	0	0	0.0560818	0.4131925	0.3656526	0.003333
1.10.3.12	0.4023403	0	0.159194	0	0.0709576	0.079135	0.0703052	0.126336
1.10.3.-	134.801511	92.00984	137.993373	101.889925	100.630087	96.746921	113.387537	125.657595
1.10.3.3	0	0.00135	0	0	0.0815	0.39697828	0.434	0.0004348
1.10.9.1	0.0255	0.0157	0.004	0	0	0	0	0
1.11.1.10	9.45955403	4.54516321	13.09781085	3.58976875	4.24121725	4.82521935	6.7492535	8.61956325
1.11.1.1	241.155927	235.735956	279.122586	226.299346	255.680477	239.097193	256.059198	289.410507
1.11.1.15	123.4748453	110.5912626	138.767524	117.0376212	125.471405	132.1578319	121.5223286	125.5366504
1.11.1.19	0.384625	0.7969655	3.236376	0.8750445	1.946315	0.6875945	1.902281	0.9932455
1.11.1.21	1.19893216	0.24719998	0.29958453	0.1231389	0.27069382	0.97350132	0.97425908	0.07258029
1.11.1.5	16.7772639	28.6022054	33.7320509	21.168069	37.12123868	22.4706708	35.3648553	43.9516072
1.11.1.-	5.66026495	8.19410294	10.43360169	4.41497195	13.89339972	6.60222175	7.22122039	9.69293531
1.11.1.6	65.4878746	61.7997682	98.9028208	45.5285036	70.4225022	38.3755921	71.6346532	90.1056836
1.11.1.7	8.0079316	2.108568	14.650886	3.085646	3.2879541	3.8591515	5.5344093	3.877936
1.11.1.9	119.306288195	105.07390945	96.0193147	103.9684442	92.6088492	109.037203455	104.3400153	85.2895606
1.11.2.4	2.19196138	0.80350532	0.26556076	0.12665078	0.14470937	0.07852318	0.18011384	0.03810522
1.12.1.2	59.2651692	54.219792	9.294764	51.130746	23.767469	60.374116	36.189392	0.66047
1.12.1.3	0.7602156	0.0232112	0.3143613	1.1192685	0.3728613	0.9283536	0.30485	0.0234
1.12.1.4	0.3787074	0	0	0	0	0	0	0
1.12.5.1	4.192563	3.743696	5.074306	5.209417	8.183801	5.973564	2.405719	0.720587
1.12.7.2	70.3372773	60.1545795	20.88844	61.4816765	37.109361	80.7259695	48.1468355	4.5527415
1.12.98.1	0.04466844	0.0035	0	0	0.0385	0.27	0.257	0.015
1.12.99.-	0	0	0.024	0	0	0	0	0
1.12.99.6	24.3620149	10.019944	37.386466	17.875758	25.25585	25.553048	22.2769495	16.910159
1.13.11.11	8.2474266	5.216699	5.971628	0.373278	0.2791794	1.968052	1.7201088	2.029654

| Back to Page: →1← |

C. Sample ‘Pathway Abundance Profile’ generated using Global Mapper module of Vikodak.

A snapshot of the ‘Pathway Abundance Profile’ generated using Global Mapper module of Vikodak.

A tab-delimited file is generated, wherein first column represents various pathways inferred and first row represents various samples of the environment (corresponding to the input file).

Features	Sample1	Sample2	Sample3	Sample4	Sample5	Sample6	Sample7	Sample8	Sample9
ABC_transporters	1175.35	1265.71	1076.2	1062.96	1118.84	1064.83	1133.96	1196.14	1261.27
Acridone_alkaloid_biosynthesis	1.39048	0.02955	0.0525	0.013	0	0.0214734	0.006	0.015544	0.1195
Adipocytokine_signaling_pathway	631.819	518.962	611.651	542.073	514.536	571.093	552.461	493.356	518.313
Aflatoxin_biosynthesis	303.066	336.708	314.43	272.897	328.185	294.688	323.699	330.273	323.895
Alanine_aspartate_and_glutamate_metabolism	6908.7	6566.49	6371.26	6121.85	6268.26	6499.51	6468.76	6073.6	6781.04
alpha-Linolenic_acid_metabolism	1136.22	994.71	1192.81	903.394	998.306	961.4	1045.89	983.185	959.894
Aminoacyl-tRNA_biosynthesis	5415.56	5537.56	5173.39	5299.91	5303.58	5282.21	5348.13	5320.25	5698.61
Aminobenzoate_degradation	3719.7	3522.22	3560.29	3226.47	3360.43	3376.25	3503.75	3323.62	3672.1
Amino_sugar_and_nucleotide_sugar_metabolism	11247.6	11698.5	10751.2	10332.2	10920.4	10053.4	10948.6	12256.9	10697
Anthocyanin_biosynthesis	1761.18	1760.68	1599.97	1662.93	1675.13	1710.81	1684.31	1590.74	1943.75
Antigen_processing_and_presentation	2.64001	1.30574	4.78232	1.55169	6.87689	3.20137	3.29455	1.87011	2.99879
Arachidonic_acid_metabolism	765.274	751.915	819.708	684.64	753.428	671.323	793.807	798.122	689.317
Arginine_and_proline_metabolism	8341.65	8159.68	8449.41	7437.41	8110.01	7627.16	8155.63	8353.15	7595.13
Ascorbate_and_aldarate_metabolism	2533.99	2724.84	2877.53	2183.12	2742.59	1845.07	2568.18	3428.28	1745.26
Atrazine_degradation	369.497	358.324	445.579	334.949	352.32	302.682	375.831	415.466	319.42
Bacterial_chemotaxis	372.208	407.11	379.48	303.619	383.47	252.739	385.105	496.22	220.177
Bacterial_invasion_of_epithelial_cells	203.713	199.562	204.365	197.437	185.645	191.283	196.731	187.958	211.177
Bacterial_secretion_system	2023.47	1984.29	1767.28	1906.03	1806.48	1980.27	1837.35	1672.17	2342.01
Basal_transcription_factors	552.566	547.559	482.527	520.564	523.586	545.662	536.886	500.871	576.529
Base_excision_repair	2202.38	2197.22	2180	2071.02	2159.95	2133.4	2160.3	2110.02	2274.78
Benzoate_degradation	3850.88	3370.85	3895.29	3050.05	3228.07	3139.75	3488.49	3400.81	3114.74
beta-Alanine_metabolism	1969.9	1809.47	2056.28	1731.41	1856.78	1758.09	1907.49	1869.54	1666.12
beta-Lactam_resistance	131.375	180.918	182.474	146.372	172.49	114.819	181.608	238.258	111.475
Betalain_biosynthesis	1291.36	1273.45	1261.1	1183.51	1261.89	1184.6	1268.7	1286.85	1213.27
Biosynthesis_of_12-_14-_and_16-membered_macrolides	1349.08	1352.44	1205.31	1280.62	1262.79	1318.08	1272.68	1181.72	1550.5
Biosynthesis_of_ansamycins	1677.11	1707.12	1561.93	1614.84	1600.79	1652.98	1606.13	1570.7	1895.17
Biosynthesis_of_siderophore_group_nonribosomal_peptides	389.252	384.762	386.431	370.874	376.764	332.761	389.825	411.265	328.474

| Back to Page: →1← |

D.Sample 'Pathway class' and 'super-class' files generated using the Global Mapper module of Vikodak

(i) A snapshot of the 'Pathway class' abundance profile generated using the Global Mapper module of Vikodak. (ii) A snapshot of the 'Pathway super-class' abundance profile generated using the Global Mapper module of Vikodak.

A tab-delimited file is generated, wherein first column represents various (i) pathway classes (ii) pathway super-classes inferred and the first row represents various samples of the environment (corresponding to the input file).

(i)

Features	Sample1	Sample2	Sample3	Sample4	Sample5	Sample6	Sample7	Sample8	Sample9	Sample10
Amino_acid_metabolism	51616.9	48730.9	51510.2	45095.7	48083.7	46825	48923.7	48062.9	47065.8	48318.3
Biosynthesis_of_other_secondary_metabolites	20389.1	19807.7	19573.5	18622.9	19139	19164.8	19427.9	19025.4	20382.8	19073.1
Cancers	5820.11	5693.31	5902.99	5235.45	5528.91	5284.15	5640.98	5824.16	5292.79	5849.24
Cancers__Specific_types	836.603	881.448	912.64	819.977	842.184	775.465	872.406	919.369	808.86	925.374
Carbohydrate_metabolism	84696.6	86159.5	85080.7	77313.9	82901.4	75833.7	83381.8	90926.7	78054.3	91115.3
Cell_communication	562.977	590.899	638.402	556.263	602.718	522.143	593.946	661.371	506.176	663.082
Cell_growth_and_death	2643.05	2680.11	2482.34	2508.62	2586.27	2520.13	2609.62	2633.94	2642.6	2636.05
Cell_motility	1224.22	1229.95	1049.26	1115.59	1107.82	1103.61	1124.31	1122.07	1282.28	1126
Chemical_structure_transformation_maps	20.1611	8.19892	20.9698	12.8314	23.4739	16.0264	14.4334	4.53681	6.28021	5.02347
Digestive_system	6047.44	5924.11	5907.46	5612.03	5678.76	5606.55	5891.25	6018.87	5677.14	6039.04
Endocrine_and_metabolic_diseases	1082.5	1094.29	1399.37	1050.99	1293.74	1006.29	1246.47	1323.53	726.251	1334.86
Endocrine_system	3654.33	3300.72	3516.12	3219.76	3108.46	3326.56	3331.09	3150.25	3357.81	3183.36
Energy_metabolism	21762.7	20930.6	21369.3	19922.8	20584.9	20637.3	21046.9	20131.9	21385.3	20225
Excretory_system	238.227	223.452	241.337	236.018	217.242	208.418	228.173	229.828	218.53	231.345
Folding_sorting_and_degradation	5591.5	5405.68	5228.25	5298.44	5304.91	5436.96	5357.54	5108.62	5721.91	5117.01
Glycan_biosynthesis_and_metabolism	12293	12451.7	11893	12234.3	12362.2	12475.1	12504	12358.4	12887.3	12360.3
Immune_diseases	472.641	451.547	475.772	438.037	433.156	432.427	444.023	455.986	446.595	457.488
Immune_system	974.901	1068.05	1165.52	976.195	1054.42	898.056	1077.45	1230.72	848.727	1235.49
Infectious_diseases__Bacterial	7034.96	7027.41	6621.1	6672.55	6625.23	6498.36	6710.13	6665.6	7269.09	6695.14
Infectious_diseases__Parasitic	739.746	627.229	591.289	698.634	602.064	719.963	616.297	525.625	781.055	529.196
Lipid_metabolism	23209.6	22527.8	22976.6	20448.3	21384.1	20706.6	22275	22218.3	21821.8	22362.2
Membrane_transport	4563.38	4894.43	4370.42	4180.9	4512.77	3956.96	4402.02	5014.4	4545.02	5008.37
Metabolism_of_cofactors_and_vitamins	25360	24390.6	24236.2	23529.7	23666.9	24417.4	24137.6	23117.7	25462.1	23176.9
Metabolism_of_other_amino_acids	12015.8	11486.6	11279.3	10966.7	10991.3	11256.3	11305.3	10879.4	11984	10923.8
Metabolism_of_terpenoids_and_polyketides	17532.5	17208.4	16578.6	15960.9	16415.3	16301.3	16707.7	16191.7	17932.8	16248.8
Nucleotide_metabolism	21358.7	21274.5	20939.5	20419.6	20803.6	20315.7	21067.7	21381.9	20525.9	21398

(ii)

Features	Sample1	Sample2	Sample3	Sample4	Sample5	Sample6	Sample7	Sample8	Sample9	Sample10
Cellular_Processes	10198.9	9883.71	9547.69	9362.97	9288.38	9525.31	9636.16	9407.79	10248	9456.91
Environmental_Information_Processing	8064.16	8378.67	7402.89	7497.87	7681.14	7329.88	7659.27	8055.06	8554.07	8059.07
Genetic_Information_Processing	23250.4	23031.5	21815.2	22107	22266.7	22686.7	22481.4	21630.7	24366.9	21652.6
Human_Diseases	15986.6	15775.2	15903.2	14915.6	15325.3	14716.7	15530.3	15714.3	15324.6	15791.3
Metabolism	322465	314736	317710	291555	304864	295275	310746	314406	305171	315587
Organismal_Systems	11150.8	10752.4	11070.1	10282.3	10281.8	10247	10764.6	10879.9	10318.2	10940.5

| Back to Page: →1← |

E. Sample 'individual functional contributions' file generated using Independent Contributions sub-module of Global Mapper

A snapshot of the Pathway Abundance Profile(s) of individual microbial taxa of an environment (environment refers to the taxonomic abundance data provided as input to Global Mapper), generated using 'Independent Contributions' sub-module of Global Mapper.

A tab-delimited file is generated, wherein first column represents various taxa present in the environment and the first row represents various pathways inferred in each of the taxa.

Features	Oxidative_phosphorylation	Vibrio_cholerae_	Betalain_biosynthesis	Phosphotransferase_system_PTS	Tyrosine_metabolism	RNA_polymerase	Flagellar_assembly
Treponema	0.04	0.04	0.04	0.04	0.04	0.14	0.12
Veillonella	7.00	3.67	5.51	3.11	8.05	23.53	11.95
Brachybacterium	0.82	0.64	0.61	0.67	1.16	3.01	1.67
Brevundimonas	0.02	0.01	0.01	0.00	0.01	0.02	0.02
Campylobacter	3.99	2.59	4.43	0.00	3.91	8.18	7.48
Capnocytophaga	3.33	1.63	3.71	0.69	3.82	7.03	7.30
Cardiobacterium	0.00	0.00	0.00	0.00	0.00	0.00	0.00
Catonella	0.04	0.01	0.03	0.17	0.04	0.07	0.14
Caulobacter	0.00	0.00	0.00	0.00	0.00	0.00	0.00
Cellulosimicrobium	0.00	0.00	0.00	0.00	0.00	0.00	0.00
Cellvibrio	0.00	0.00	0.00	0.00	0.00	0.00	0.00
Centipeda	0.00	0.00	0.00	0.00	0.00	0.00	0.00
Chryseobacterium	0.00	0.00	0.00	0.00	0.00	0.00	0.00
Acidovorax	0.00	0.00	0.00	0.00	0.00	0.00	0.00
clostridiumsensustricto	0.00	0.00	0.00	0.00	0.00	0.00	0.00
ClostridiumXIVa	0.00	0.00	0.00	0.00	0.00	0.00	0.00
Comamonas	0.30	0.34	0.38	0.07	0.25	0.52	0.36
Acinetobacter	2.04	0.86	1.99	0.63	1.49	2.46	3.27
Corynebacterium	2.14	1.01	1.87	1.25	1.50	6.16	4.50
Cryptobacterium	0.00	0.00	0.00	0.00	0.00	0.00	0.00
Cupriavidus	0.00	0.00	0.00	0.00	0.00	0.00	0.00
Actinobacillus	0.00	0.00	0.00	0.00	0.00	0.00	0.00
Deinococcus	0.01	0.01	0.01	0.00	0.01	0.03	0.02
Dermacoccus	0.00	0.00	0.00	0.00	0.00	0.00	0.00
Desulfobulbus	0.00	0.00	0.00	0.00	0.00	0.00	0.00
Abiotrophia	0.05	0.02	0.05	0.00	0.04	0.05	0.14
Actinomyces	2.82	1.25	1.35	2.03	2.08	3.06	4.37
Dialister	0.15	0.05	0.09	0.04	0.14	0.28	0.32
Dietzia	0.00	0.00	0.00	0.00	0.00	0.00	0.00

| Back to Page: →1← |

F. Sample 'Core-Pathway' results generated using Global Mapper module of Vikodak

A snapshot of the results generated through Core-Pathway analysis option provided in Global Mapper module of Vikodak.

A tab-delimited file is generated, wherein first column represents the pathways that have been attributed as "Core" and second column represents the bootscore for each pathway deduced as core. Bootstrap score serves as an index of confidence for core-prediction. Higher the bootstrap score, the higher is the confidence of core-prediction. The bootstrap score can range between 0-100.

Core_Pathway(s)	Bootstrap_Score
Bacterial_chemotaxis	100
Bacterial_secretion_system	100
Base_excision_repair	100
beta-Lactam_resistance	100
Biosynthesis_of_siderophore_group_nonribosomal_peptides	100
Biosynthesis_of_type_II_polyketide_backbone	100
Cell_adhesion_molecules_CAMs	100
Citrate_cycle_TCA_cycle	100
DNA_replication	100
ECM-receptor_interaction	100
Flagellar_assembly	100
Lipopolysaccharide_biosynthesis	100
Mismatch_repair	100
Nonribosomal_peptide_structures	100
Other_glycan_degradation	100
Pathogenic_Escherichia_coli_infection	100
Phosphotransferase_system_PTS	100
Protein_export	100
RNA_polymerase	100
Sulfur_relay_system	100
Synthesis_and_degradation_of_ketone_bodies	100
Bootstrap Score (0-100); s=70% of total samples; n=1000	
s: No. of samples randomly picked	
n: Number of iterations performed	

| Back to Page: →1← |

G. Sample 'representative functional abundance' data generated by Global Mapper module of Vikodak

A snapshot of the sample 'representative functional abundance data' that can be generated using either of the 'mean, median or relative contribution' as central tendency in Global Mapper module of Vikodak.

A tab-delimited file is generated, where in first column represents the 'name of the function (pathway/ class/ super-class)' and second column represents the 'effective' abundances of each of the functions computed using the mean/median/relative contribution of the functional abundance profile for all samples of the environment.

Feature	Abundance
Butirosin_and_neomycin_biosynthesis	710.357
RNA_polymerase	559.4
Photosynthesis	542.46
Flagellar_assembly	536.82
Synaptic_vesicle_cycle	536.82
Phagosome	536.812
Phosphotransferase_system_PTS	516.138
Drug_metabolism_-_cytochrome_P450	497.078
Metabolism_of_xenobiotics_by_cytochrome_P450	472.726
Cell_cycle	425.368
Anthocyanin_biosynthesis	385.803
Carbohydrate_digestion_and_absorption	340.033
Vibrio_cholerae_infection	332.443
Dioxin_degradation	325.678
Biosynthesis_of_12-_14-_and_16-membered_macrolides	315.775
Maturity_onset_diabetes_of_the_young	315.035
Glycosaminoglycan_biosynthesis_-_keratan_sulfate	309.758
Mucin_type_O-Glycan_biosynthesis	309.758
Cytosolic_DNA-sensing_pathway	292.644
Circadian_rhythm	292.317
Huntingtons_disease	292.208
Retinol_metabolism	288.77
Type_I_polyketide_structures	284.033
MAPK_signaling_pathway	282.823
Bacterial_secretion_system	268.452
Proximal_tubule_bicarbonate_reclamation	268.181
HIF-1_signaling_pathway	265.671
Insulin_signaling_pathway	256.927
Flavonoid_biosynthesis	251.189
Stilbenoid_diarylheptanoid_and_gingerol_biosynthesis	249.63

| Back to Page: →1← |

H. Sample Input and Output files of ISFA module of Vikodak

A snapshot representing (i) Sample input for the Inter Sample Feature Analyzer (ISFA) module of Vikodak. A tab-delimited 'function abundance data' file containing the names of the features (functions) in first column, names of the samples in second row and names of the classes (categories) to which those samples belong to (viz. diseased or healthy) in first row (ii) Sample output/result file of ISFA module. First row of the tab-delimited file contains the names of the differentiating features between a given pair of sample classes and second row contains the bootstrap score assigned to each differentiating feature.

A bootstrap score of 100 would mean that the given feature was identified as Significantly Differentiating (at or below user specified p-value) in all (100%) iterations.

(i)

Class	ClassA	ClassA	ClassA	ClassA	ClassA	ClassB	ClassB	ClassB	ClassB	ClassB	ClassB
Features	SampleA1	SampleA2	SampleA3	SampleA4	SampleA5	SampleB1	SampleB2	SampleB3	SampleB4	SampleB5	SampleB6
ABC_transporters	8602.59	12225.3	13059.9	10535.6	8419.82	9516.3	13631.9	15085.3	11505.7	19507.8	12165.6
Acridone_alkaloid_biosynthesis	101.64	0.875	34.8866	82.1286	2.625	1.125	0.151812	1	0.783562	1.6875	0
Acute_myeloid_leukemia	10655.7	12708.9	16223.3	12818.8	9942.71	9827.94	14259.2	18694.4	30261.4	18786.4	13182.5
Adherens_junction	8136.01	11251.8	13442.8	11337.1	10019.1	9872.1	13246	13126.5	17199.3	18969.3	13587.5
Adipocytokine_signaling_pathway	13856.7	16622.5	20773.8	17912.9	12515.8	13336.1	19307.1	22946.6	17010.1	27657.1	18461.5
Adrenergic_signaling_in_cardiomyocytes	14265.7	22358	26125.7	17729.1	12901.9	14043.5	21115.9	24149.5	18166	33575.2	18878.1
Aflatoxin_biosynthesis	19677.8	27728.6	26447.3	19979.3	18522.8	19459.1	28229.7	31573.8	24960.2	40294.9	25642
African_trypanosomiasis	1131.75	3607.09	2531.85	2917.72	1720.92	1382.42	3749.93	1348.14	671.14	4292.09	1441.75
Alanine_aspartate_and_glutamate_metabolism	18940.2	24373.3	27878.3	22383	17734.6	18918.9	26964.6	30086.9	24441.9	40132.8	26304.4
Alcoholism	9078.2	12985.2	13164.6	11194.5	9458.38	9945.64	13013.9	15434.5	16292	16966.1	13694.7
Aldosterone-regulated_sodium_reabsorption	9802.85	16408.1	19218.5	12050	9129.36	9218.34	14295.4	15945.7	16859.7	24178	12171.3
alpha-Linolenic_acid_metabolism	8558.5	11874.4	12315.1	10142.6	7893.98	7818.36	11642.9	13207.1	10250.4	16405.9	12910.5
Alzheimers_disease	18021.3	24561.5	29160.7	23133.7	17813	18078	27818.3	30394.1	25179.1	38882.9	23286.7
Aminoacyl-tRNA_biosynthesis	29827.1	37644.2	43697.9	35810.6	27194.9	30860.6	43829.5	49774.5	37267.8	61084.6	40897.5
Aminobenzoate_degradation	11128.4	16294.7	14911.9	12423	9989.4	11211.7	16320.6	17969.9	14720.7	21755.2	16017.7
Amino_sugar_and_nucleotide_sugar_metabolism	16668.8	24246.7	25919.3	21094.5	16774.3	17901.4	27111.8	27680.9	21432.1	37064.8	23401.8
Amoebiasis	14742.8	18428.2	21671.9	19169.6	12960.3	18778.6	22241.9	32306.7	24037	27172.2	20598.5
Amphetamine_addiction	13071.2	19424.8	18162.3	15153.3	11353.1	13206.3	17838.5	21906.7	16159.8	24862.7	17585.4
Amyotrophic_lateral_sclerosis_ALS	4367.05	8908.05	7952.7	7287.98	5172.23	6169.44	10523.2	6820.27	4787.58	15329.2	5959.1
Anthocyanin_biosynthesis	80208.1	108329	118279	91284.8	75594.3	81684.3	114917	129809	104300	161620	115894
Antigen_processing_and_presentation	442.723	301.662	406.006	261.103	261.572	101.409	242.175	184.528	448.127	1015.9	571.714
Apoptosis	10127.3	16014	16106.1	12864.4	10404.5	10176	16222.4	16370.4	15865.9	22196.7	14339.1
Arachidonic_acid_metabolism	6263.64	11751.9	10107.7	8999.77	7216.31	6430.2	10927.4	11887.9	8009.88	13980.2	7816.83
Arginine_and_proline_metabolism	11583.3	15967.9	17133.8	14232.2	11776.3	12284.2	18795.3	19936.4	15826.2	27023.5	16077.1
Ascorbate_and_aldarate_metabolism	7501.58	13669.6	11649.3	10712.4	8242.3	9059.08	14491.9	11060.5	8257.77	19103.3	9618.57
Asthma	207.782	212.191	146.953	223.228	208.528	74.4992	267.847	115.902	254.413	465.803	152.551
Atrazine_degradation	9064.1	9647.41	10666.9	9199.43	7957.17	7071.23	11695.5	12662.9	9253.55	17266.5	11276.9
Axon_guidance	9110.77	12597	14535	11695.1	9044.5	8797.35	14360.9	14717	15874	20413.9	11451.7
Bacterial_chemotaxis	9948.98	13438.9	13834.7	13013	8440.95	7548.93	17367.5	14069.2	11045.7	19196.1	10209.2

(ii)

Differentiating Features	Bootstrap Score
Aflatoxin_biosynthesis	100
alpha.Linolenic_acid_metabolism	100
Aminoacyl.tRNA_biosynthesis	100
Arachidonic_acid_metabolism	100
Ascorbate_and_aldarate_metabolism	100
beta.Lactam_resistance	100
Biosynthesis_of_siderophore_group_nonribosomal_peptides	100
Biosynthesis_of_type_II_polyketide_backbone	100
Biosynthesis_of_unsaturated_fatty_acids	100
Brassinosteroid_biosynthesis	100
Butanoate_metabolism	100
Butirosin_and_neomycin_biosynthesis	100
Carbon_fixation_in_photosynthetic_organisms	100
Cell_cycle_._Caulobacter	100
Chlorocyclohexane_and_chlorobenzene_degradation	100
D.Glutamine_and_D.glutamate_metabolism	100

| Back to Page: →1← |

I. Sample input files for Local Mapper module of Vikodak

A snapshot of sample input file for Local Mapper module of Local Mapper containing (i) samples pertaining to a single category (ii) samples pertaining to more than one category.

Input files are required to be in tab-delimited format, such that the first row of the file(s) contains the name(s) of sample category(ies), second row consists of sample names and first column contains the names of the bacterial genera.

It is pertinent to note here that Local Mapper enables comparison between different sample categories(classes) if more than one category of samples are provided as input.

(i)

Class	SampleA	SampleA	SampleA	SampleA	SampleA	SampleA	SampleA	SampleA	SampleA	SampleA	SampleA
Features	SampleA1	SampleA2	SampleA3	SampleA4	SampleA5	SampleA6	SampleA7	SampleA8	SampleA9	SampleA10	SampleA11
Abiotrophia	0	8	19	0	69	0	23	0	0	0	6
Acetobacter	0	0	0	0	0	0	0	0	0	0	0
Achromobacter	0	0	0	0	0	0	0	0	0	0	0
Acidovorax	0	0	0	0	0	0	1	0	0	0	1
Acinetobacter	28	1	6	22	5	4	1	3	21	6	0
Actinobacillus	0	0	0	0	0	0	0	0	0	0	3
Actinomyces	19	96	93	126	52	30	59	27	27	186	30
Aerococcus	0	0	0	0	0	0	0	0	0	0	0
Aeromonas	3	1	1	3	0	0	0	0	0	0	0
Aestuariimicrobium	0	0	0	0	0	0	0	0	0	0	0
Aggregatibacter	4	189	10	0	20	56	19	21	0	31	195
Agreia	0	0	0	0	0	0	0	0	0	0	0
Alcanivorax	0	0	0	0	0	0	1	0	0	0	0
Alkanindiges	0	0	0	0	0	0	0	0	0	0	0
Allisonella	0	0	0	0	0	0	0	0	0	0	0
Alysiella	0	0	0	0	0	0	0	0	0	0	0
Amaricoccus	0	0	0	0	0	0	0	0	0	0	0
Anaerobacter	0	0	0	0	0	0	0	0	0	0	0
Anaerococcus	0	0	0	0	0	0	0	0	0	0	0
Anaeroglobus	12	3	16	21	2	0	74	4	11	0	0
Anaerovorax	0	0	0	12	0	5	2	0	0	0	0
Aquincola	0	0	0	0	0	0	0	6	0	0	1
Armatimonas/Armatimonadetes_gp1	0	0	0	0	0	0	0	0	0	0	0
Arthrobacter	0	0	4	0	0	0	0	0	0	0	0
Asteroleplasma	0	0	0	0	0	0	1	0	0	0	0
Atopobium	10	0	42	11	0	1	9	5	6	3	1
Azorhizobium	0	0	0	0	0	0	0	0	0	1	0
Bacillariophyta	0	0	0	0	0	0	0	0	0	0	0
Bacillus	0	0	0	0	0	0	0	0	0	0	0

(ii)

Class	SampleA	SampleA	SampleA	SampleA	SampleA	SampleA	SampleA	SampleB	SampleB	SampleB	SampleB	SampleB	SampleB
Features	SampleA1	SampleA2	SampleA3	SampleA4	SampleA5	SampleA6	SampleA7	SampleB1	SampleB2	SampleB3	SampleB4	SampleB5	SampleB6
Abiotrophia	0	8	19	0	69	0	23	0	0	0	6	0	0
Acetobacter	0	0	0	0	0	0	0	0	0	0	0	0	0
Achromobacter	0	0	0	0	0	0	0	0	0	0	0	0	0
Acidovorax	0	0	0	0	0	0	1	0	0	0	1	1	0
Acinetobacter	28	1	6	22	5	4	1	3	21	6	0	15	1
Actinobacillus	0	0	0	0	0	0	0	0	0	0	3	0	0
Actinomyces	19	96	93	126	52	30	59	27	27	186	30	35	22
Aerococcus	0	0	0	0	0	0	0	0	0	0	0	0	0
Aeromonas	3	1	1	3	0	0	0	0	0	0	0	0	0
Aestuariimicrobium	0	0	0	0	0	0	0	0	0	0	0	0	0
Aggregatibacter	4	189	10	0	20	56	19	21	0	31	195	110	0
Agreia	0	0	0	0	0	0	0	0	0	0	0	0	0
Alcanivorax	0	0	0	0	0	0	1	0	0	0	0	0	0
Alkanindiges	0	0	0	0	0	0	0	0	0	0	0	0	0
Allisonella	0	0	0	0	0	0	0	0	0	0	0	0	0
Alysiella	0	0	0	0	0	0	0	0	0	0	0	0	0
Amaricoccus	0	0	0	0	0	0	0	0	0	0	0	0	0
Anaerobacter	0	0	0	0	0	0	0	0	0	0	0	0	0
Anaerococcus	0	0	0	0	0	0	0	0	0	0	0	0	0
Anaeroglobus	12	3	16	21	2	0	74	4	11	0	0	0	51
Anaerovorax	0	0	0	12	0	5	2	0	0	0	0	2	0
Aquincola	0	0	0	0	0	0	0	6	0	0	1	1	0
Armatimonas/Armatimonadetes_gp1	0	0	0	0	0	0	0	0	0	0	0	0	0
Arthrobacter	0	0	4	0	0	0	0	0	0	0	0	0	0
Asteroleplasma	0	0	0	0	0	0	1	0	0	0	0	0	0
Atopobium	10	0	42	11	0	1	9	5	6	3	1	0	13

| Back to Page: →1← |

J. Sample output files generated by Local Mapper module of Vikodak

A snapshot of the sample output file generated by Local Mapper module of Vikodak containing (i) abundance values of various enzymes present in a given metagenomic environment corresponding to the user specified metabolic pathway (ii) a 'user data mapping' file pertaining to the enzymes of the user specified pathway, which can be uploaded at the corresponding KEGG pathway link at www.genome.jp/kegg/pathway.html to obtain a visual depiction of relative abundances of various enzymes of the given pathway in the metagenomic environment(s). The aforesaid visual depiction may be referred to in Supplementary Fig 3.

(i)

Elements	ClassA	ClassB
1.1.1.1	1294.147	1574.679
1.1.1.3	410.923	571.094
2.1.2.1	236.121	253.541
4.4.1.1	155.805	130.216
2.5.1.47	145.731	152.327
1.1.1.95	132.638	134.155
4.2.1.20	132.027	163.753
4.3.1.17	131.633	149.583
2.7.2.4	119.601	110.877
4.4.1.2	113.699	121.023
3.1.3.3	113.258	128.265
1.8.1.4	109.472	151.213
4.1.2.5	107.659	89.386
4.2.3.1	105.368	111.608
4.3.1.19	103.364	122.007
1.2.1.3	102.295	198.967
4.1.1.20	91.425	94.049
1.2.1.11	90.42	96.521
2.1.1.20	87.615	81.332
2.7.8.8	87.331	77.562
2.7.1.39	86.953	106.194
5.4.2.12	73.771	79.597
1.1.1.29	70.247	70.691
2.7.1.31	65.768	85.498
1.4.4.2	61.439	64.652
4.3.1.18	51.047	55.64
2.6.1.52	49.624	62.537

(ii)

EC	ColorCode
1.1.1.1	#ff6699
1.1.1.3	#ffefcc
2.1.2.1	#ffefcc
4.4.1.1	#ffefcc
3.5.1.-	#ffefcc
2.5.1.47	#ffefcc
1.1.1.95	#ffefcc
4.2.1.20	#ffefcc
4.3.1.17	#ffefcc
2.7.2.4	#ffefcc
1.1.1.-	#ffefcc
4.4.1.2	#ffefcc
3.1.3.3	#ffefcc
1.8.1.4	#ffefcc
4.1.2.5	#ffefcc
4.2.3.1	#ffefcc
4.3.1.19	#ffefcc
1.2.1.3	#ffefcc
4.1.1.20	#ffefcc
1.2.1.11	#ffefcc
2.1.1.20	#ffefcc
2.7.8.8	#ffefcc
2.7.1.39	#ffefcc
5.4.2.12	#ffefcc
2.6.1.-	#ffefcc
1.1.1.29	#ffefcc
2.7.1.31	#ffefcc
1.4.4.2	#ffefcc
4.3.1.18	#ffefcc

| Back to Page: →1← |
