# Supplementary material for: Vikodak - A Modular Framework for Inferring Functional Potential of Microbial Communities from 16S Metagenomic Datasets
Source: PLoS One. 2016 Feb 5;11(2):e0148347. doi: 10.1371/journal.pone.0148347 (PMC4746064; doi:10.1371/journal.pone.0148347)
Supplement: S1 Table — A tabulated summary of various datasets employed to validate the utility of various modules of Vikodak. (DOCX) [file pone.0148347.s009.docx]

**S1 Table: Summary of various datasets employed for validation of Vikodak**

A tabulated summary of various datasets employed to validate the utility of various modules of

Vikodak

| **Sample ID** | **Site** | **Datasets** | **Reference** |
| --- | --- | --- | --- |
| Gut HMP | Gut  (Human) | 306 | Consortium THMP, 2012 [11] |
| Gut Prebiotics | Gut  (Human) | 283 | Kato et al., 2014 [12]  Xiao et al., 2014 [13] |
| Oropharynx, American  Sputum | Oral  Cavity  (Human) | 13 | Botero et al., 2014 [14] |
| Chinese Sputum | Oral  Cavity  (Human) | 55 | Cui et al., 2012 [15] |
| Skin | Skin  (Human) | 149 | Alekseyenko et al., 2013 [16] |
| Sub-gingival | Oral  Cavity  (Human) | 91 | Griffen et al., 2012 [17] |
| Vaginal | Vagina  (Human) | 394 | Romero et al., 2014 [18] |
| Amazonian soil | Soil | 18 | DDBJ ID: ERA411828 |
| Nematode | *Litoditis*  *marina* | 36 | DDBJ ID: SRP064694 |
